# Genome-wide investigations reveal the population structure and selection signatures of Nigerian cattle adaptation in the sub-Saharan tropics

**DOI:** 10.1186/s12864-022-08512-w

**Published:** 2022-04-15

**Authors:** David H. Mauki, Abdulfatai Tijjani, Cheng Ma, Said I. Ng’ang’a, Akanbi I. Mark, Oscar J. Sanke, Abdussamad M. Abdussamad, Sunday C. Olaogun, Jebi Ibrahim, Philip M. Dawuda, Godwin F. Mangbon, Rudovick R. Kazwala, Paul S. Gwakisa, Ting-Ting Yin, Yan Li, Min-Sheng Peng, Adeniyi C. Adeola, Ya-Ping Zhang

**Affiliations:** 1 State Key Laboratory of Genetic Resources and Evolution & Yunnan Laboratory of Molecular Biology of Domestic Animals, Kunming Institute of Zoology, Chinese Academy of Sciences, Kunming, China; 2 Sino-Africa Joint Research Center, Chinese Academy of Sciences, Kunming, China; 3 Kunming College of Life Science, University of Chinese Academy of Sciences, Kunming, China; 4 Faculty of Pharmaceutical Sciences, Chinese Academy of Sciences, Shenzhen Institute of Advanced Technology, Shenzhen, Guangdong China; 5 International Livestock Research Institute (ILRI), Addis Ababa, Ethiopia; 6 Centre for Genomics Research and Innovation, National Biotechnology Development Agency, Abuja, Nigeria; 7 Ministry of Agriculture and Rural Development, Secretariat, Ibadan, Nigeria; 8 Taraba State Ministry of Agriculture and Natural Resources, Jalingo, Nigeria; 9 Department of Animal Science, Faculty of Agriculture, Bayero University, Kano, Nigeria; 10 Department of Veterinary Medicine, University of Ibadan, Ibadan, Nigeria; 11 Department of Veterinary Surgery and Theriogenology, College of Veterinary Medicine, University of Agriculture Makurdi, Makurdi, Nigeria; 12 Division of Veterinary Office, Serti, Nigeria; 13 Faculty of Veterinary Medicine, Sokoine University of Agriculture, Morogoro, Tanzania; 14 Department of Microbiology, Parasitology and Biotechnology/ Genome Science Center, Sokoine University of Agriculture, Morogoro, Tanzania; 15 State Key Laboratory for Conservation and Utilization of Bio-Resources in Yunnan, School of Life Sciences, Yunnan University, Kunming, China; 16 Centre for Biotechnology Research, Bayero University, Kano, Nigeria; 17 Center for Excellence in Animal Evolution and Genetics, Chinese Academy of Sciences, Kunming, 650223 China

**Keywords:** Cattle, Genotyping-by-sequencing, Genome, Convergent evolution, Africa

## Abstract

**Background:**

Cattle are considered to be the most desirable livestock by small scale farmers. In Africa, although comprehensive genomic studies have been carried out on cattle, the genetic variations in indigenous cattle from Nigeria have not been fully explored. In this study, genome-wide analysis based on genotyping-by-sequencing (GBS) of 193 Nigerian cattle was used to reveal new insights on the history of West African cattle and their adaptation to the tropical African environment, particularly in sub-Saharan region.

**Results:**

The GBS data were evaluated against whole-genome sequencing (WGS) data and high rate of variant concordance between the two platforms was evident with high correlated genetic distance matrices genotyped by both methods suggestive of the reliability of GBS applicability in population genetics. The genetic structure of Nigerian cattle was observed to be homogenous and unique from other African cattle populations. Selection analysis for the genomic regions harboring imprints of adaptation revealed genes associated with immune responses, growth and reproduction, efficiency of feeds utilization, and heat tolerance. Our findings depict potential convergent adaptation between African cattle, dogs and humans with adaptive genes *SPRY2* and *ITGB1BP1* possibly involved in common physiological activities.

**Conclusion:**

The study presents unique genetic patterns of Nigerian cattle which provide new insights on the history of cattle in West Africa based on their population structure and the possibility of parallel adaptation between African cattle, dogs and humans in Africa which require further investigations.

**Supplementary Information:**

The online version contains supplementary material available at 10.1186/s12864-022-08512-w.

## Background

African indigenous cattle are considered to be the most desirable livestock by small scale farmers in the continent due to their vast economic benefits. These ranges from meat, milk, drought power, source of leather, manure and bride price. The origin of the domesticated cattle can be traced to around 10,000 years before present (YBP) in Southwest/South Asia, and in West Asia for indicine and taurine cattle, respectively [1, 2], before their migration to other parts of the globe. In Africa, the earliest group of cattle known to have migrated into the continent were the *Bos taurus taurus* circa 7,000 – 4,000 YBP and later the *Bos taurus indicus* circa 4,000 – 2,000 YBP from their domestication centers [3, 4].

A large number of African taurine are found in West Africa [5]. However, few taurine cattle are also present in other parts of Africa, for instance the taurine Sheko from Ethiopia in East Africa [6] with majority of them currently considered to be crossbreds [5] as compared to other types of African taurine particularly the Muturu breed of West Africa [7]. Several cattle population in Nigeria are a mixture of both taurine and zebu ecotypes [5, 8]. They are suggested to have been introduced circa 1,400 YBP [3, 9] from eastern Africa which is hypothesized to be the entry point of zebus in Africa from Asia during the Indian-ocean maritime trade [10].

In the past decade, the advancement of genomic technologies has made it possible to analyze the genomic DNA of individuals using WGS and GBS [11, 12]. The GBS technology involves the use of restriction enzymes (REs) to select targeted polymorphic genomic regions for reduced representation [11], lowering genome associated data complexes, and sequencing costs [13], thereby enabling large numbers of individuals to be sequenced [14]. A comparative performance ratio of GBS relative to WGS in mammals such as cattle has not yet been fully investigated, although a similar approach has been applied in pigeon birds [15]. However, the assessments of GBS and SNP chip panels have been reported in plants and animals [16–19].

In this study, we evaluate the efficacy of datasets generated by GBS versus WGS and the subsequent application in downstream population genetics analyses. To the best of our knowledge, we applied GBS approach for the first time in assessing the genetic diversity and adaptation mechanism of cattle samples from Nigeria. Several studies have been carried out to analyze the population structure and selection signatures of modern African cattle [7, 20], however, the genomes of cattle samples from Nigeria has not been intensively explored. Our findings present the unique genetic patterns of Nigerian indigenous cattle in sub-Saharan West Africa.

## Results

### Comparative analysis based on newly generated data from five selected Nigerian samples

The 5-sample dataset generated a total of 924,152 and 23,955,622 GBS and WGS SNPs, respectively, of high-quality filtered genotypes (more than 95% genotype call rate) for comparisons (Additional file 1: Figures S1 and S2). However, 462,823 variants equivalent to 49.4% of all the total loci called by GBS were reliable for evaluation. Out of these, 93.05% equivalent to 430,635 variants were found in common with WGS dataset while 430,574 of them were concordant at a 99.99% concordance rate i.e., similar alternative alleles at the same loci with WGS dataset (Additional file 1: Figure S1; Additional file 1: Figure S3). Aside from this, GBS dataset showed elements of novelty such that a proportion of 6.95% (32,188 sites) of all the variant loci had partial novel sites since 3% of them (987 sites) still overlapped with WGS dataset leaving 97% (32,201 sites) completely novel i.e., do not overlap with WGS dataset. On the other hand, we have unfortunately observed a major shortcoming in GBS technology. Despite the fact that the detected variants (single nucleotide polymorphisms, SNPs) were highly concordant with WGS, genotype matches between GBS and WGS were unsatisfactorily low and not consistent, ranging between 92.8% and 52.1% in the calling of heterozygous (RA) and homozygous non-reference (AA) genotypes, respectively(see details in in Additional file 1 containing the Supplementary Notes). Such a relatively low rate of genotype matches is not an unexpected scenario for low sequencing coverage data.

### The merged GBS and published WGS datasets for population genetics analyses

**Fig. 1:**
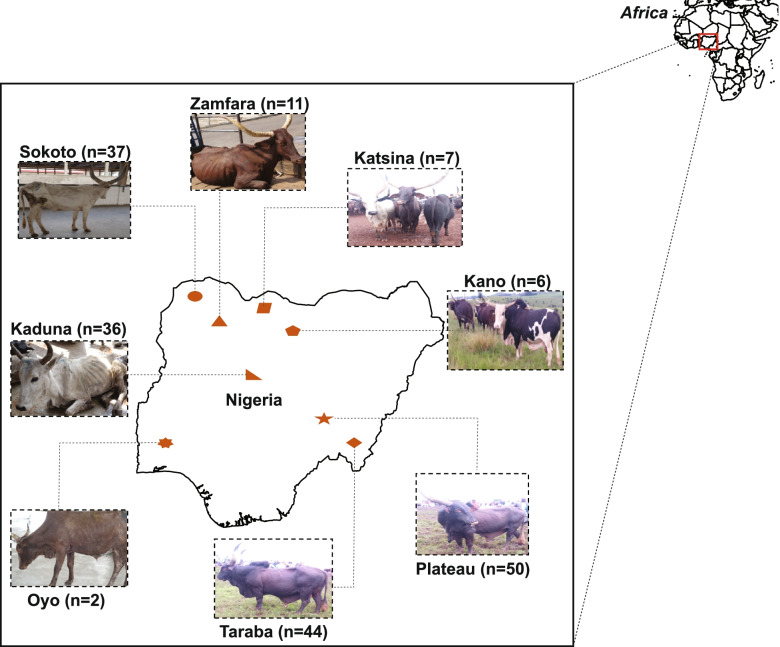
Geographical distribution of indigenous cattle in Nigeria. The figure shows sampling locations and photographs of indigenous cattle from Nigeria. The sample size in each sampling location was as follows: Kaduna (*n* = 36), Kano (n = 6), Katsina (*n* = 7), Oyo (*n* = 2), Plateau (*n* = 50), Sokoto (*n* = 37), Taraba (*n* = 44), and Zamfara (*n* = 11)

The genomes of 193 cattle sampled from Nigeria (Fig. 1) were sequenced to generate ~ 1.1 billion clean reads with an average of ~ 4.87 × depth coverage that ranged between 4.33 to 6.28 reads per individual (Additional file 2: Table S1). The reads were then aligned to the taurine reference genome (*B. taurus* UMD 3.1) at an average mapping rate of 99.13% and jointly merged and genotyped with 75 publicly available genomes [20–23] (Additional file 1: Table S2). After merging our dataset of the 193 Nigerian cattle genomes containing 3,282,427 SNPs with the additional 75 genomes containing 22,197,616 SNPs, a total of 268 cattle samples with 649,577 common biallelic SNPs of high genotyping rate (0.93), was retained for downstream analyses. The published cattle samples used in this study were classified according to their ecotypes and geographical locations as follows: African taurine (including 8 Muturu and 7 N’Dama), African humped cattle (included 10 Boran, 8 Kenana, and 9 Ogaden), African Sanga (12 Ankole), European taurine (7 sampled Holstein breed individuals) and Asian cattle samples (5 pure Asian zebu and 3 Asian *B. indicus* X *B. taurus* crossbreeds) and the outgroup samples (represented by 5 *B. javanicus* and one *Bubalus bubalis*) (Additional file 1: Figure S4).

### Population structure and genetic diversity of Nigerian cattle

**Fig. 2:**
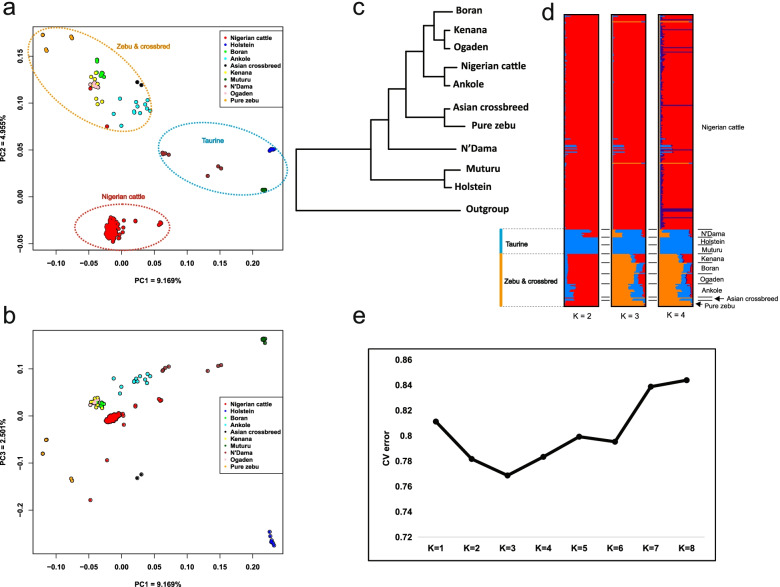
Population structure and evolutionary relationship. PCA plotted by using R software with PC1 against PC2 (**a**) and PC1 against PC3 (**b**). Colours represent cattle populations from different geographical regions as described in Additional file 2: Table S1 and Additional file 1: Table S2. Here, PCA was constructed using samples from Europe (Holstein, blue), Asia for both pure Asian (orange) and crossbreed zebu (black) and Africa (Boran—green, Ankole—cyan, Kenana—yellow, Muturu—dark green, N'Dama—brown, Ogaden – pink and Nigeria). Nigerian cattle are in red. NJ phylogenetic tree of the relationships between Nigerian cattle and all other populations used in this study (**c**). Here the concatenated tree was constructed using *B. bubalis* as outgroup. The proportion of ancestry admixture for each individual’s genome assuming different number of ancestral populations (*K* = 2, 3 and 4) (**d**). The admixture plot indicates three possible clusters of cattle: the Nigerian cattle (red), the taurine (light blue) and the zebu and their crossbred (orange). The cross-population error plot shows optimum ancestral populations for inferring genetic admixture is at *K* = 3 (**e**)

The structure of the cattle populations was extrapolated by principal component analysis (PCA), admixture and phylogenetic tree. PCA results depict three major clusters of cattle (Fig. 2a) and showed agreement of the three major lineages of cattle (Fig. 2b) that include the European *B. taurus* (blue), the African *B. taurus* (represented by Muturu in green and N’Dama in brown) and Asian *B*. *indicus* (orange) [24, 25]. The clustering of Nigerian cattle together with East African zebu and at intermediate position between pure *B. taurus* and Asian *B*. *indicus* possibly suggest the admixed (*B. taurus* X *B*. *indicus*) background of the majority of African zebu cattle [7] and that Nigerian cattle could be more of the zebu background well supported by paternal marker studies [5, 26]. Principal Component (PC)1 explained 9.169% of the total variation, separates all zebu cattle populations from taurine; European (Holstein) and African (N’Dama and Muturu) taurine in Fig. 2a while PC2 which explained 4.955% of the total variation depicts geographical partitioning between the Nigerian and East African cattle populations. Noticeably, while the distinction between Nigerian cattle and other populations captured in PC2 on Fig. 2a may be affected by the genotyping effect of GBS, it is consistent with previous maternal studies [26]. PC1 and PC2 extrapolate those three clusters of cattle observed in this study as follows: first, all zebu together with their crossbred clustered together, the second cluster contained only the taurine cattle, and third is Nigerian cattle appearing as a distinct cluster. The concatenated neighbour joining (NJ) phylogenetic tree plotted using the 193 GBS samples and all the reference populations has given a consistent story that Nigerian cattle are of crossbred background having clustered in the same clade with Ankole (Fig. 2c).

Generally, PCA depicts the unique genetic constitution of Nigerian zebu cattle from other African zebu or African hybrids (Additional file 1: Figure S4) clearly supported by the admixture plot (Fig. 2d), reflecting an important genetic resource reservoir [8]. Furthermore, the unsupervised clustering approach results (Fig. 2d) indicate *K* = 3 as the optimum ancestral populations for inferring the genetic structure and admixture (Fig. 2e) which corresponds to the three cattle clusters. Both admixture and PCA analyses inferred the population of Nigerian zebu cattle is homogenous lacking clear genetic structure albeit with minimal levels of admixture.

**Fig. 3:**
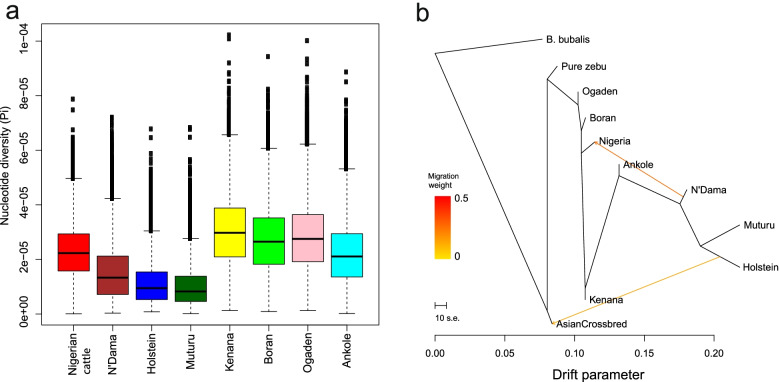
Diversity of African cattle and gene flow between cattle populations. The genetic diversity estimated by nucleotide diversity index (Pi) in non-overlapping windows of 100 kb window size (**a**). Pattern of population splits and migration between Nigerian and other cattle populations (**b**). The tree shows migration or evidence of gene flow from other populations particularly the N’Dama breed into the gene pool of Nigerian cattle population. Boran, Kenana, Ogaden represent East African zebus; N’Dama, and Muturu represent the African taurine and Ankole also known as Sanga breed, is the hybrid between African zebu and taurine; pure zebu and Asian crossbred represent cattle from Asia and East Africa; the Holstein breed stands as the only European taurine cattle used in this study; and Nigeria represent cattle sampled in Nigeria; *B. bubalis* is the outgroup (more details are given in Additional file 1: Table S2)

The genetic diversity results are shown in Fig. 3a. Nigerian zebu cattle together with all zebu cattle from Africa depict high genetic diversity compared to taurine cattle populations which is not unexpected [7, 20]. Muturu, indigenous Nigerian taurine cattle among other taurine showed the lowest level of genetic diversity.

### Genetic admixture

Maximum likelihood (ML) tree was constructed by TreeMix software in order to infer evidence of gene flow between cattle populations from Europe, Asia and Africa using sequentially migration edges (1 to 4) (Fig. 3b; Additional file 1: Figures S5-6). Our findings showed the possibility of gene flow from N’Dama, the African taurine cattle breed towards Nigerian zebu cattle and from European taurine to Asian cattle in two steps migration event. Nonetheless, at the migration edge 4, we also observe a possible gene flow between Nigerian and European cattle. Furthermore, we also assessed the introgression between Nigerian cattle and other cattle populations from different geographical settings complementing TreeMix results as displayed in Additional file 1: Table S3. We used the nonparametric ‘ABBA-BABA’ test to examine the exchange of genetic variation between two divergent populations from their individual genomes in particularly to study the admixture in Nigerian zebu cattle genomes. Our findings showed significant evidence of admixture for three population pairs involving the Nigerian zebu cattle exclusively: European taurine and Nigerian zebu cattle, African taurine and Nigerian zebu cattle, and East African zebu and Nigerian zebu cattle. These findings are coherent with TreeMix analysis and also complemented by population structure which showed admixture in Nigerian zebu cattle at rather low degree (Fig. 2d). Due to the geographical proximity between Nigerian cattle and the African taurine, hybridization between the two cattle populations is definitely eminent [27, 28].

### Signatures of selection

Signatures of selection was computed by two different approaches: the composite likelihood ratio (CLR) approach implemented in SweeD and PBS (Additional file 2: Tables S4-8) using UMD3.1 reference genome and the concordance with ARS-UCD1.2 genome assembly was assessed afterwards (Table 1). For CLR test, focusing on only Nigerian zebu cattle, we obtained 240 positively selected genes (PSGs) in the 1% threshold level (Additional file 2: Table S4). We also used PBS to compute for genes that are under selection in Nigerian zebu cattle by testing different computational scenarios (Additional file 2: Tables S5-8). In all scenarios, Nigerian zebu cattle were considered as the target population while *B. bubalis* and *B. javanicus* were the outgroups.

**Table 1:** Candidate regions of selection in Nigerian cattle identified by both PBS and CLR approaches in the 1% windows analysis using UMD3.1 and ARS-UCD1.2 bovine reference genome assemblies

**Genome Assembly**	**BTA**	**PBS**		**CLR**		**Genes identified**
		**Region/Position (Mb)**	**PBS value**	**Region/Position (Mb)**	**CLR**	
UMD3.1	1	24.55—24.56	0.5074	71.79—130.27	11.0443—35.1499	*ROBO2, IL12A, DLG1, PIK3CB*
ARS-UCD1.2	1	24.55—134.97	0.7934—0.8605	71.79—130.27	11.0443—35.1499	*AMOTL2, FILIP1L, LOC101906248, GBE1, ROBO2, IL12A, DLG1, PIK3CB*
UMD3.1	2	-	-	112.21	23.6732	*SERPINE2*
ARS-UCD1.2	2	-	-	112.21	23.6732	*SERPINE2*
UMD3.1	3	-	-	26.85	24.1543	*ATP1A1*
ARS-UCD1.2	3	-	-	26.85	24.1543	*ATP1A1*
UMD3.1	5	18.34	0.5601	-	-	*KITLG*
ARS-UCD1.2	5	18.34	0.5601	-	-	*KITLG*
UMD3.1	6	70.22—70.31	0.356—0.5339	-	-	*KIT, LOC112447081*
ARS-UCD1.2	6	5.61—109.69	0.5801—0.6146	-	-	*KIT, LOC112447081, RAB28, LOC112447028, LOC112447126, LOC112447030, LOC112447169, LOC112447029, GABRG1, LOC112447030*
UMD3.1	7	21.7	0.4454	-	-	*IL4*
ARS-UCD1.2	7	-	-	-	-	-
UMD3.1	11	-	-	49.08	30.2696	*ATOH8*
ARS-UCD1.2	11	-	-	49.08	30.2696	*ATOH8*
UMD3.1	22	11.6—11.61	0.5781	-	-	*ACAA1, MYD88*
ARS-UCD1.2	22	-	-	-	-	-
UMD3.1	23	7.1—51.82	0.3904—0.4787	-	-	*BOLA-DYB, BOLA-DOA*
ARS-UCD1.2	23	7.11	0.5991	-	-	*BOLA-DYB*

**Fig. 4:**
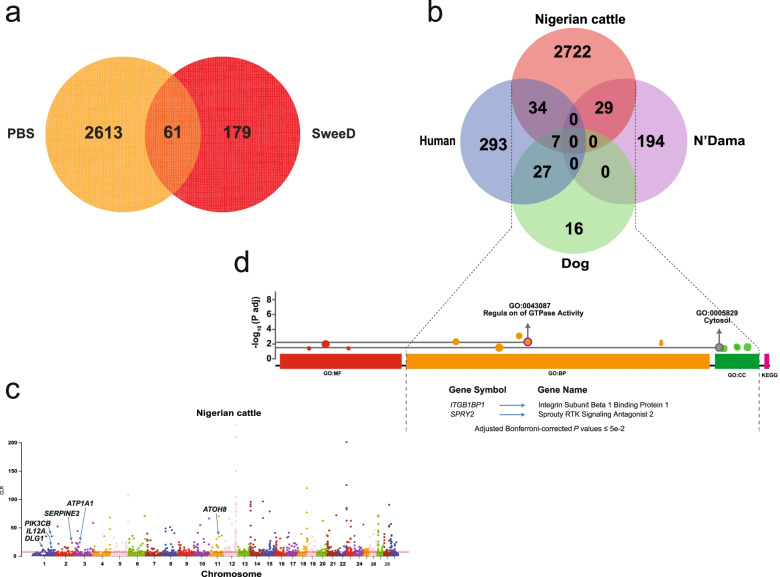
Plots showing results for signatures of selection. Venn diagram shows the unique and the shared PSGs identified in candidate regions under selection by both PBS and SweeD in Nigerian cattle (**a**). An amount of 2613 and 179 PSGs refer to the private genes detected by PBS and SweeD, respectively. And 61 PSGS are the common genes in both PBS and SweeD. Overlap of PSGs between African human, cattle (Nigerian cattle and N’Dama) and dog populations (**b**). The PSGs in African human, N’Dama, and dogs except the Nigerian cattle, were adopted from published publicly available datasets [42–44]. For Nigerian cattle the uniquely detected PSGs by PBS and SweeD combined together (a total of 2792 genes) excluding the 61 common genes were used. This information is described elsewhere in the results section and in the legend part (**a**). Manhattan plot indicates the autosomal genomes in Nigerian cattle generated by SweeD (**c**). The functional enrichment results of the overlapping PSGs between African human, cattle, and dog populations (**d**). Here, the gene ontology (GO) display three categories of biological function namely: MF, molecular function; BP, biological processes; and CC, cellular components [75]. Additionally, KEGG, Kyoto Encyclopedia of Genes and Genomes biological pathways were also integrated [76]

Using PBS approach in scenario one (PBS 1), 2027 PSGs were detected in Nigerian zebu cattle following their separation from the common ancestor with European cattle (Additional file 2: Table S5). In the second category (PBS 2), 2029 PSGs were identified in Nigerian zebu cattle following the divergence from their Asian zebu counterpart (Additional file 2: Table S6). We then carried out a third scenario (PBS 3) by comparing Nigerian zebu cattle against cattle from both Europe and Asia (Euro-Asian), where 2021 PSGs were detected (Additional file 2: Table S7). The final scenario (PBS 4) was carried out to determine evidence of domestication signatures in Nigerian zebu cattle in relation to other African cattle populations of zebu lineage, as such, 2031 PSGs were contemporarily unveiled (Additional file 2: Table S8). In all these four scenarios, PBS generated a total of 2674 PSGs in Nigerian cattle (Fig. 4a and Additional file 2: Tables S5-8). Since the contrasted groups (Nigerian cattle exclusively as the target population against other populations) are genotyped by different methods (GBS and WGS), the estimation of allele frequencies between populations through PBS procedures was importantly addressed by using the intersected genomic regions that would limit false positives. The merging of the two datasets was done at the very beginning of the data analysis prior to downstream analyses as described in the methodology section “Data merge”. Nonetheless, using the same five (5) samples, the estimated alleles genotyped by both methods either by GBS or WGS depicted high correlation (*r* = 0.9999355, *p*-value = 6.22e-07, Pearson's product-moment correlation test) supporting the viability of GBS data. Basically, the windows of the 1% threshold level from PBS and CLR test included in total 2674 and 240 PSGs, respectively, indicating that 2613 and 179 (totaling 2792 PSGs) were unique to PBS and CLR, respectively, whereas 61 of all the detected PSGs overlapped in both analyses (Fig. 4a). Moreover, these private genes (2792 PSGs) obtained between CLR and PBS in Nigerian cattle genomes were further utilized to understand the possibility of convergent adaptation between African cattle, dogs and humans (Fig. 4b) co-existing in similar West African environment.

Functional enrichment analysis was then conducted to determine the biologically enriched pathways in Nigerian zebu cattle (Table 2 and Additional file 2: Tables S9-13). Among the 61 overlapping PSGs detected by both PBS and CLR, the gene *IL12A* was highly overrepresented or expressed more in biological pathways which could be associated with physiological roles related to the immune system of the host (Table 2). The latter gene and *LAMA4* are possibly associated with the immunity against African trypanosomiasis, the endemic tropical cattle disease in tsetse infested areas. Furthermore, the functional enrichment analysis using the 240 selective genes detected by CLR test analysis alone showed evidence of 17 annotated PSGs which could be involved in biological processes, KEGG and cellular components’ pathways (Additional file 2: Table S9). Among these genes are three protein-coding genes *IL12A* [29]*, DLGI* [30] and *PIK3CB* [31] related to the host immune and *ATOH8 which could be* linked to reproduction [32] (Table 1 and Fig. 4c). Two of the other protein-coding genes *could be* linked to more than one trait, which include *ATP1A1* known to play a role in efficiency of feeds utilization and tolerance to environmental thermal stresses [33, 34] and *SERPINE2* [35] associated with growth and development of skeletal muscles [36] and also possibly involved in immune regulation of the host through release of immunoglobulins [37]. Notably, PBS functional enrichment analyses using the gene list in Additional file 2: Tables S5-8 revealed several signals of positive selection (Additional file 2: Tables S10-13) likely associated with important local environmental adaptations (Table 1 and Additional file 1: Figures S7, S8, S9 and S10). These included genes that offer the host’s immune against tropical parasitic diseases such as the African trypanosomiasis, a common disease in cattle known to infect many of the zebu cattle breeds (trypano-susceptible) than the taurine cattle (trypano-tolerant) which possess the disease’s resistance mechanism. An example of this gene is *MYD88* [38] (Table 1 and Additional file 1: Figure S7 and S8). In this category, we also found *ROBO2* gene which has been previously reported to provide immune response against Newcastle disease in chicken [39]. Other important genes detected includes those related to the regulation of developmental processes (GO:0,050,793) such as coat colour phenotypic traits, for instance *KITLG* and *KIT* [20, 40]. Some of the genes present in 1% windows threshold were also linked to resistance to tick infestation (*SPAST* [7] and *BOLA* [20]) and control of *Plasmodium falciparum* (*IL4* [41]) (Additional file 1: Figure S8).

**Table 2:** Functional enrichment of the common Nigerian cattle candidate genes identified by both CLR and PBS at empirical 99^th^ percentile threshold level

Source of GO category	Category description	GO term	Adjusted *P* value*	Gene name
GO:MF	interleukin-17 binding	GO:0,019,975	0.019626	*IL12A*
GO:MF	interleukin-12 beta subunit binding	GO:0,042,163	0.019626	*IL12A*
GO:MF	interleukin-27 binding	GO:0,045,513	0.019626	*IL12A*
GO:MF	interleukin-12 binding	GO:0,019,972	0.039253	*IL12A*
GO:CC	interleukin-12 complex	GO:0,043,514	0.022269	*IL12A*
KEGG	African trypanosomiasis	KEGG:05,143	0.043979	*IL12A, LAMA4*

^*^P values are Bonferroni-corrected at *P* values ≤ 5e-2

Moreover, we unraveled the occurrence of candidate genes putatively involved in convergent selection in African human and domestic animals such as cattle and dogs by comparing the enrichment output of the overlapping genes between our 2792 unique PSGs (Fig. 4a) and published gene lists for African humans [42], N’Dama, the African taurine [43] and African dogs [44] (Fig. 4b). Notably, in computing for the evidence of convergent selection, both the shared (61) and the unique (2792) gene lists were employed. However, only the unique genes could unveil the common genes under selection among species with 97 PSGs common to at least 2 species (Fig. 4b).

GO enrichment analysis using the 41 overlapping annotated genes between African cattle (Nigerian zebu cattle) and humans among which 7 overlapped also with dogs (Fig. 4b, revealed significant enriched biological pathways (Fig. 4d). Following this procedure, one of the enriched PSGs *ITGB1BP1* identified, when blasted on BGVD (Bovine Genome Variation Database) [45] revealed a GO term’s description: regulation of GTPase activity which confers to a common biological pathway between African cattle, humans and dogs as previously speculated [42, 44]. *SPRY2* PSG was also found as an evidence of common ways of adaptation between African N’Dama (*B. taurus* lineage) [43] and Nigerian zebu cattle (*B. indicus* lineage) when enrichment analysis was performed using their 29 overlapping PSGs (Fig. 4b) depicting a similar GO description: regulation of GTPase activity and also a similar GO:0,005,829 term (Fig. 4d) as reported previously in African humans [42].

## Discussion

### Evaluation of Genotyping-by-sequencing

To explore the potential of GBS approach, we applied it to understand the population genomics of Nigerian zebu cattle through the analysis of genetic structure, and signatures of selection. We firstly generated optimum evidence of its applicability on cattle genomic studies by comparing it with WGS. We obtained high rates of concordance for the variants calls (SNPs) between GBS and WGS datasets (Additional file 1: Figure S11), which exhibited similar patterns of genetic structure (Additional file 1: Figure S12) even though they differed in their allelic distribution (Additional file 1: Figure S13) and had low genotypes matching rate (putatively associated with the low sequencing depth of GBS, Additional file 2: Table S1). Based on the concordance of the variant calls, GBS could be reliable for its applicability in cattle genomics studies [46], even if its use without imputation may compromise the estimation of some genetic parameters [47, 48]. Consequently, we noticed that GBS in some instances failed to yield accurate genetic variation estimate as observed in PC 1 (Additional file 1: Figure S12a), where the five Nigerian cattle individuals typed by GBS cluster closer to Ankole than to WGS Nigerian genotypes. This genotyping effect is coherent with the low proportion of genotype match between GBS and WGS.

### Genetic variation and evidence of introgression in Nigerian zebu cattle

Our study also revealed that the genetic diversity of Nigerian zebu cattle similarly to other zebus in Africa is higher compared to taurine as previously speculated [20]. Both versions of the references genomes depicted similar pattern of the genetic diversity (Additional File 1: Figure S14). The slight degree of admixture observed by the admixture analysis may have been mediated by the gene flow from other cattle populations such as the taurine N’Dama, as evidenced by TreeMix and introgression analyses. These two cattle populations (Nigerian cattle and N’Dama) occupy the same geographical region of West Africa hence hybridization between the two is eminent. Despite the low degree of admixture detected in cattle from Nigeria, they still clustered as a homogenous population with lack of genetic structuring as recently observed in studies based on matrilineal genetic markers [26] and bovine high density SNP data [24]. The lack of genetic structuring observed in Nigerian cattle could be possibly due to productivity and fitness selective pressures [26] a similar scenario previously observed in Borgou breed, a bovine hybrid population from Benin [27]. Figure 1 shows some morphological differences in Nigerian cattle. However, despite these morphological disparities many of them display no structure probably due to lack of genetic differentiation. In some instances, some of the Nigerian cattle individuals show more of the similarities with taurine (indicated in blue) in the admixture plot (Fig. 2d) at optimum *K* = 3. Taken altogether our findings suggest evidence of introgressed taurine alleles into the gene pool of Nigerian zebu cattle.

Farmers in West Africa prefer crossing of African taurine and zebu in order to formulate a crossbreed cattle popularly known as Méré that possess combined genetic attributes of both disease tolerance and production traits [20, 27]. However, introgression from African taurine to Nigerian zebu cattle has not been fully established based on our *D*-statistics analysis (Additional file 1: Table S3). Zebu cattle have long been considered the African dairy and/or beef cattle [49] due to their high levels of milk production and large body size adopted for meat production and draught adaptive traits [6, 28, 50]. The African taurine for instance the Muturu and N’Dama cattle are known for their small size [23, 45] a feature that confers their low body size as compared to zebu cattle albeit they possess high tolerance to enzootic diseases such as trypanosomiasis and dermatophilosis prevalent in the Sub-humid region of West Africa and also less susceptible to tick‐borne diseases compared to zebu [51]. Furthermore, a phylogenetic concatenated NJ tree (Additional file 1: Figure S15) using the five WGS samples (Additional file 2: Table S14), supports closer relationship with zebus (by clustering closer to Boran), which could result from crossbreeding, as suggested by the proximity with Ankole, the well-known crossbred population (Fig. 2c). On the other hand, the high genetic diversity or variation scenario observed in African cattle populations including the Nigerian cattle has been reported consistently throughout the African continent based on matrilineal and autosomal genetic analyses [26, 27, 52]. Notably, the lack of genetic structure or low genetic differentiation observed in the current study, as is the majority of African cattle populations [26], reflects the random mating of cattle populations in Africa with low practice of artificial selection as compared to breeding practices in other regions such as Europe [43].

### Domestication impacts and adaptation in sub-Saharan tropics

The investigation of signatures of selection for the Nigerian zebu cattle was to elucidate and update information on the adaptive traits of cattle in the tropics particularly in the sub-Saharan region of West Africa. In most cases tropical environments are usually characterized by diseases, poor forage, high temperatures, exposure to ultraviolet and inappropriate management policies which are mostly observed in developing countries [44, 53]. Previous studies have shown that modern humans and domesticated animals share imprints of evolution in their genomes acquired during domestication especially when occupying sympatric geographical regions [44, 54]. Some important genes such as *ADGRE1* [44] and *ASIP* [55] have been identified to be involved in the evolution of both human and dogs or water buffalo and domestic cattle, respectively. In this study, we also identified other genes commonly involved in the evolution of African cattle, dogs and human.

Our study observed that Nigerian zebu cattle facing similar environmental challenges common in West Africa such as trypanosomiasis have also contemporarily developed similar immune response like the African taurine against these prevailing challenges [7, 56]. PBS and CLR test approaches have both indicated PSGs including *IL12A*, *LAMA4*, *MYD88*, *SPAST* and *BOLA* to confer resistance mechanisms towards the African trypanosomiasis and tick infestation [7, 20, 29, 38]. Nonetheless, several other PSGs such as *IL4* associated with the control of malaria, one of the most prevalent tropical diseases in Africa [41] was also observed in this study conferring its role of malaria resistance in Nigerian zebu cattle genomes presently in West Africa. The African continent is characterized with its unique adverse conditions such as high temperatures. Nigerian zebu cattle in particular may have also developed adaptability mechanism towards such conditions such as *KITLG* and *KIT* [20, 40] which control coat colour phenotypes and the regulation of physiological temperature in the tropics possibly in a similar mutual fashion with the hair cell differentiation and blood circulation observed in Chinese zebu cattle [22]. Nonetheless, it is worth to mention some of the genes such as *ECI1* and *RNPS1* present in the highest 1% PBS value of the outlier windows (Additional file 2: Tables S5-8). Based on their physiological function information retrieved from BGVD (Bovine Genome Variation Database) [45], these genes are related to metabolism of both catabolic and anabolic processes.

Notably, in the attempt to detect signatures of selection using GBS approach, it has further extended the evidence of concordance between WGS and GBS. Our study observed that GBS detected similar imprints of adaptation such as *KIT* on BTA 6 [20], *SPAST* on BTA 11 [7] and *BOLA* on BTA 23 [20] as previously unveiled by WGS data.

To disclose the possibility of shared aspects of domestication or convergent adaptation between African human, cattle and dogs present in West African part of the continent, regions of PSGs from humans [42], and dogs [44] were compared with our Nigerian zebu cattle dataset and N’Dama, the African taurine cattle [43] for their possible common physiological functions. When comparing PBS to CLR (SweeD), PBS computation was conducted with less stringent threshold yielding a huge evidence of PSGs imprinted in Nigerian cattle genomes, even if we cannot exclude some degree of false positives. Some of the identified PSGs conferring to convergent aspects of adaptation include *ITGB1BP1* (Integrin Subunit Beta 1 Binding Protein 1) and *SPRY2* (Sprouty RTK Signaling Antagonist 2) found to overlap between Nigerian zebu cattle, humans and N’Dama, the African taurine (Fig. 4d). The *SPRY2* gene is important in embryo development in African taurine [43], and it is also involved in the regulation of GTPase activity together with the *ITGB1BP1*. We speculate that these two genes may have similar biological function with the *ADGRE1* gene which is as well involved in GTPase regulator activity in African dogs [44] as it is in African humans [42]. The shared physiological function of GTPase in cattle, dogs and human confers to the probable shared evolutionary aspects with African humans in playing defensive mechanism towards Malaria as previously unveiled [42]. Notably, this hypothetical narrative may hold true since these three species co-exist in tropical environments hinting at their possible shared evolutionary aspects. We therefore, suggest further investigation of the disease immune mechanism associated with the *SPRY2* and *ITGB1BP1* PSGs towards Malaria.

## Conclusions

This study reports the current genetic status and new insights on the adaptation of zebu cattle in sub-Saharan region of West Africa using GBS approach. The unique population structure of Nigerian zebu cattle observed serves as an important genetic resource in West Africa. We discovered the possibility of parallel or convergent adaptation among African human and domestic animals and that Nigerian zebu cattle might have acquired disease tolerance traits endemic in West Africa like their African taurine counterpart. Our study tried to investigate whether the identified PSGs in Nigerian cattle could result from convergent selection with other species occupying the same environmental conditions with regard to tropical diseases, high temperatures, scarcity of water and even of forages to mention a few. However, our finding may be speculative due to a number of reasons for instance the low sequencing coverage by GBS, or the integration of two datasets from two different genotyping platforms (only Nigerian cattle data was generated through GBS). Therefore, more efforts are still needed to determine and characterize the mechanisms of convergent adaptation in particular those conferring to resistance of diseases such as those endemics in West Africa in order to inform appropriate strategies befitting conservation, survivability of livestock, production improvement and applicability in biomedical research models for human related diseases.

## Materials and Methods

### Sample collection

Whole-blood samples (10 ml) were collected from 193 cattle coming from eight different States in Nigeria as follows (Fig. 1): Kaduna (*n* = 36), Kano (*n* = 6), Katsina (*n* = 7), Oyo (*n* = 2), Plateau (*n* = 50), Sokoto (*n* = 37), Taraba (*n* = 44), and Zamfara (*n* = 11). Genomic DNA extractions were performed following phenol–chloroform method [57] at Kunming Institute of Zoology, Chinese Academy of Sciences (CAS). The extracts were quantified using the Thermo Scientific™ NanoDrop 2000 spectrophotometer in order to assess purity of the extracted DNA. Furthermore, the DNA extracts were checked for molecular quality by running them through a 2% agarose gel against a 2 kilobase (kb) DNA ladder marker. The 193 cattle samples were sequenced using GBS platform. We further selected five samples for WGS for the evaluation of GBS and WGS platforms (Please, refer to Supplementary Notes in Additional file 1 for more details).

### Next-generation sequencing of the GBS data

Briefly, the DNA PCR extracts were then sent to Bejing Novogene (https://en.novogene.com/), where the GBS approach was carried out following the GBS protocol [11]. The GBS DNA library was prepared using 500 ng of DNA from each individual in 96-well plates before applying REs for genome reduced representation. Genomic DNA was then incubated at 37℃ with *Mse*I (New England Biolabs, NEB), T4 DNA ligase (NEB), ATP (NEB), and *Mse*I Y adapter N containing barcode. Fragment read length of 150 bases (PE150) were then sequenced using the Illumina HiSeq2500 platform with TruSeq SBS Kit v3-HS (Illumina). The whole genome-resequencing for five samples were also conducted at Beijing Novogene.

### Sequence data analysis of GBS data

Illumina sequencing GBS data for 193 cattle genomes representing a wide diversity of cattle from Nigeria in West Africa were aligned to the cattle reference UMD 3.1 assembly [58] using BWA *mem* [59] with default parameters. Picard-tools -1.119 were used to sort the reads and to remove duplicates. The Genome Analysis Toolkit (GATK v3.8) [60, 61] was used to realign indels. Subsequently, SNPs were then detected by using UnifiedGenotyper [62] integrated in GATK.

The following hard filtration criteria were carried out using GATK v3.8 for the parameters: mapping quality rank sum test (MQRankSum), Fisher strand bias (FS), quality by depth (QD), the read position rank sum test (ReadPosRankSum) and phred score (GQ). The values for each parameter were QD > 2.0, FS < 60.0, MQ > 40.0, MQRankSum > -12.5, GQ > 20, QUAL > 50.0, ReadPosRankSum > -8.0, and ((MQ0 / (1.0 * DP)) < 0.1)” > ”. After filtration, only the high quality biallelic SNPs with genotyping call rate > 90% were retained for downstream analyses. The density of SNPs in each chromosome and the allelic distribution of minor alleles are provided in Additional file 1: Figures S16 and S17, respectively.

### Concordance analysis between GBS and WGS datasets

We randomly selected five of the 193 cattle samples and re-sequenced them using WGS method in order to assess the accuracy of GBS. Common number of SNPs, and genotypes as generated by GATK were used for concordance evaluation. Notably, a Pearson correlation (r) method was used to determine the correlation between the computed distance matrices by GBS and WGS data using cor.test() test R function. More details of the evaluation assessment can be obtained in Supplementary Notes.

### Data merge

To perform population genetic analyses of the 193 genomes of Nigerian cattle GBS data, we also integrated 75 WGS genomes datasets publicly available from previous studies [20–23] representing *B. taurus* and *B. indicus* cattle of both African, European, and Asian lineages as well as *B. javanicus* and *B. bubalis* which define the outgroup. Only the overlapping genomic regions between GBS and WGS were considered using the merge parameter flag -*intersection* in GATK. Detailed information on the newly generated GBS data can be accessed in Additional file 2: Table S1. Geographical origin and other detailed information for each published cattle sample can be obtained in Additional file 1: Table S2.

### *Population genetic structure***,***admixture, and Genetic diversity*

For PCA [63], EIGENSOFT software [64] was used to generate the principal components (PCs) from the filtered autosomal biallelic SNPs which were then plotted using R software. For the main figures of both PCA and admixture we used a dataset that excluded the outgroup, only cattle populations of zebu origin, African taurines and European taurines were used. Admixture analysis was performed using the unsupervised clustering method implemented in ADMIXTURE v1.3.0 software [65] and the resulting admixture proportions were plotted in Genesis software. We also computed for the genetic distances to construct a NJ population-level phylogenetic tree using autosomal genome data constructed by PLINK v1.9 software [66] and multiple sequence alignments were performed using Clustal W v2.1 Linux version [67]. The resulting tree file was plotted by using MEGA X ver 10.1.7 software to surmise the evolutionary relationship between populations. Genetic diversity was also inferred from non-overlapping windows of 100 kb window size across the genome using VCFtools v0.1.12b software [68].

### Inference of genetic admixture

The ML tree was computed following the proposed protocol in TreeMix v1.13 software [69] in order to determine admixture events and population splits. We were only interested in understanding how the Nigerian cattle gene pool has been influenced by other cattle populations. The algorithm was run for 1 to 4 migration edges and setting the *B. bubalis* as outgroup. The outputs were plotted using the R v4.0.2 software. We furthermore tested for the introgressions between populations by performing *D*-statistics analysis for all possible combinations [70].

### Selection signature

Filtered biallelic SNPs from 193 samples were processed through VCFtools and GATK software bioinformatics tools with a genotyping rate of at least 90%, and minimum phred scaled genotype quality of 20 to produce a VCF file consisting of high-quality set of 3,282,427 SNPs. To avoid any significant false positives, the computation for the signatures of selection was done using the estimated allele frequencies from the common genomic regions between Nigerian cattle (GBS data) and other cattle populations (genotyped by WGS) merged together during PBS analyses. We used Sweep Detector (SweeD) tool that implements a composite likelihood ratio (CLR) test and the population branch statistics (PBS) [71, 72] to identify PSGs in Nigerian cattle. The CLR computation was carried out at 1000 grids (-grid 1000) to identify selective sweeps in Nigerian cattle genomes. PBS was estimated by using Wright’s *F*_*ST*_ statistics [73] in non-overlapping windows of 50 kb starting at the first variant, and in each consecutive 2 kb interval step until the last variant on each autosomal chromosome in four different approaches. In these PBS approaches, the first approach considered the European population to be the control group and the second one the cattle breeds from Asia to be the control group. Aside to this, we also combined European and Asian cattle populations as a single group called Euro-Asian to act as a control group in the third approach and the fourth approach considered the other African cattle populations of zebu descent excluding Nigerian cattle. In all scenarios Nigerian cattle were considered the target population and water buffalo and banteng were used as outgroups. We estimated changes in the allele of Nigerian cattle using PBS as follows:

$$PBS=\frac{{t}^{\mathrm{N}\_\mathrm{OT}}+ {D}^{\mathrm{N}\_\mathrm{D}}- {t}^{\mathrm{OT}\_\mathrm{D}}}{2}$$

where; PBS estimates the pairwise allele frequency (*F*_*ST*_) between Nigerian cattle (N) and other cattle populations (OT) recorded from each of the four scenarios stated above and between these populations (N and OT) and the distantly related species (D) represented herein by the outgroup samples, the water buffalo and banteng. Sequentially, the divergence time (t) of Nigerian cattle from the other populations is also determined.

### Annotation and functional enrichment

The annotation of the candidate regions was based on the *B. taurus* UMD 3.1 Gene Transfer Format file expressed by an extension (.gtf) from Ensembl release 90 [74]. Functional enrichment analysis of the annotated PSGs was conducted using a statistical overrepresentation test in g: Profiler [75] based on the Gene Ontology (GO) categories and Kyoto Encyclopedia of Genes and Genomes (KEGG) pathways [76] and the candidate gene information was also confirmed on the Bovine Genome Variation Database (BGVD) [45]. A Bonferroni-corrected–adjusted *P* value of 0.05 was used as threshold level for statistical significance. The same protocol for accessing the adaptation information imprinted in the Nigerian cattle genomes was also implemented using ARS-UCD1.2 reference genome in order to assess the reliability of our findings.

## Supplementary Information

**Additional file 1.** 

**Additional file 2.** 

## Data Availability

All new sequencing data generated in this study have been deposited in the Genome Sequence Archive (GSA) under accession number PRJCA004338. Further details are provided in Additional file 2: Table S1. Additional requests can be channeled to the corresponding authors.

## Abbreviations

BGVD: Bovine Genome Variation Database
BTA: *Bos taurus* Assembly
CAS: Chinese Academy of Sciences
CLR: Composite Likelihood Ratio
GATK: Genome Analysis Toolkit
GBS: Genotyping-by-sequencing
GO: Gene Ontology
Gtf: Gene Transfer Format
GSA: Genome Sequence Archive
KEGG: Kyoto Encyclopedia of Genes and Genomes
ML: Maximum Likelihood
NJ: Neighbor-joining
PBS: Population branch statistics
PCA: Principal component analysis
PCs: Principal components
PSGs: Positively selected genes
Res: Restriction enzymes
SweeD: Sweep Detector
WGS: Whole genome sequencing
YBP: Years before present
