# Supplementary material for: Genome-wide investigations reveal the population structure and selection signatures of Nigerian cattle adaptation in the sub-Saharan tropics
Source: BMC Genomics. 2022 Apr 15;23:306. doi: 10.1186/s12864-022-08512-w (PMC9012019; doi:10.1186/s12864-022-08512-w)
Supplement: Supplementary file 1 — Additional file 1. [file 12864_2022_8512_MOESM1_ESM.pdf]

Supplementary Information

Genome-wide investigations reveal the population structure and selection signatures of Nigerian cattle adaptation in the sub-Saharan tropics

David H. Mauki[1,2,3,4], Abdulfatai Tijjani[5,6], Cheng Ma[1,3], Said I. Ng'ang'a[1,2,3], Akanbi I. Mark[7], Oscar J. Sanke[8], Abdussamad M. Abdussamad[9], Sunday C. Olaogun[10], Jebi Ibrahim[11], Philip M. Dawuda[11], Godwin F. Mangbon[12], Rudovick R. Kazwala[13], Paul S. Gwakisa[14], Ting-Ting Yin[1], Yan Li[15], Min-Sheng Peng[1,2,3], Adeniyi C. Adeola[1,2,3,16], Ya-Ping Zhang[1,2,3,15,17]

[1] State Key Laboratory of Genetic Resources and Evolution & Yunnan Laboratory of Molecular Biology of Domestic Animals, Kunming Institute of Zoology, Chinese Academy of Sciences, Kunming, China.

[2] Sino-Africa Joint Research Center, Chinese Academy of Sciences, Kunming, China.

[3] Kunming College of Life Science, University of Chinese Academy of Sciences, Kunming, China.

[4] Faculty of Pharmaceutical Sciences, Chinese Academy of Sciences, Shenzhen Institute of Advanced Technology, Shenzhen, Guangdong, China.

[5] International Livestock Research Institute (ILRI), Addis Ababa, Ethiopia.

[6] Centre for Genomics Research and Innovation, National Biotechnology Development Agency, Abuja, Nigeria.

[7] Ministry of Agriculture and Rural Development, Secretariat, Ibadan, Nigeria.

[8] Taraba State Ministry of Agriculture and Natural Resources, Jalingo, Nigeria.

[9] Department of Animal Science, Faculty of Agriculture, Bayero University, Kano, Nigeria.

[10] Department of Veterinary Medicine, University of Ibadan, Ibadan, Nigeria.

[11] Department of Veterinary Surgery and Theriogenology, College of Veterinary Medicine, University of Agriculture Makurdi, Makurdi, Nigeria.

[12] Division of Veterinary Office, Serti, Nigeria.

[13] Faculty of Veterinary Medicine, Sokoine University of Agriculture, Morogoro, Tanzania.

[14] Sokoine University of Agriculture, Department of Microbiology, Parasitology and Biotechnology/ Genome Science Center, Morogoro, Tanzania.

[15] State Key Laboratory for Conservation and Utilization of Bio-Resources in Yunnan, School of Life Sciences, Yunnan University, Kunming, China.

[16] Centre for Biotechnology Research, Bayero University, Kano, Nigeria.

[17] Center for Excellence in Animal Evolution and Genetics, Chinese Academy of Sciences, Kunming 650223, China

Corresponding Author: Ya-Ping Zhang and Adeniyi C. Adeola

Email address: zhangyp@mail.kiz.ac.cn and chadeola@mail.kiz.ac.cn

Additional file 1: Figures S1 – 17

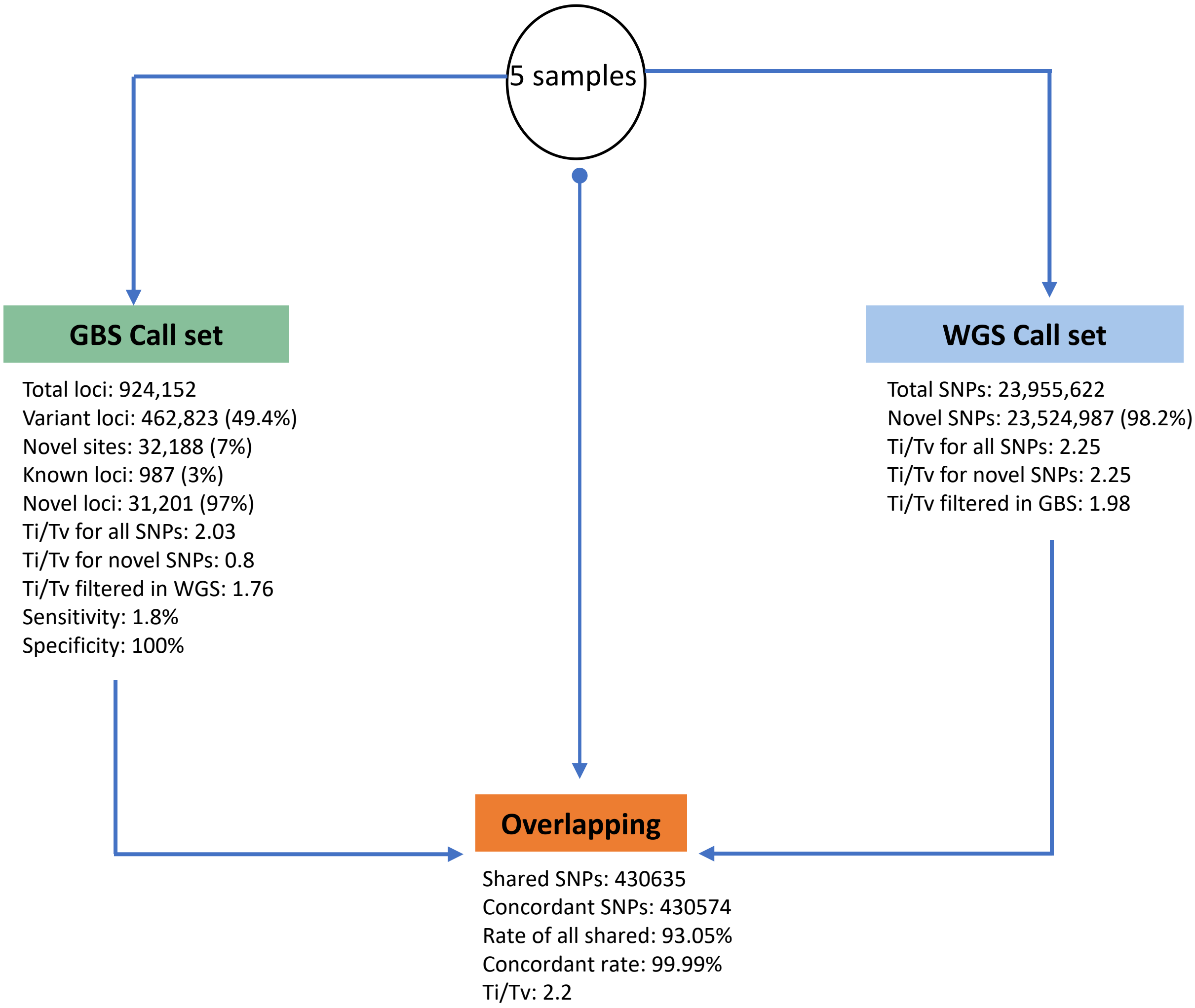

Figure S1 The performance of GBS at variant level comparatively to WGS. The statistical estimates for the rate of variant concordance, sensitivity, specificity and ti/tv ratio between GBS and WGS SNP calls are indicated.

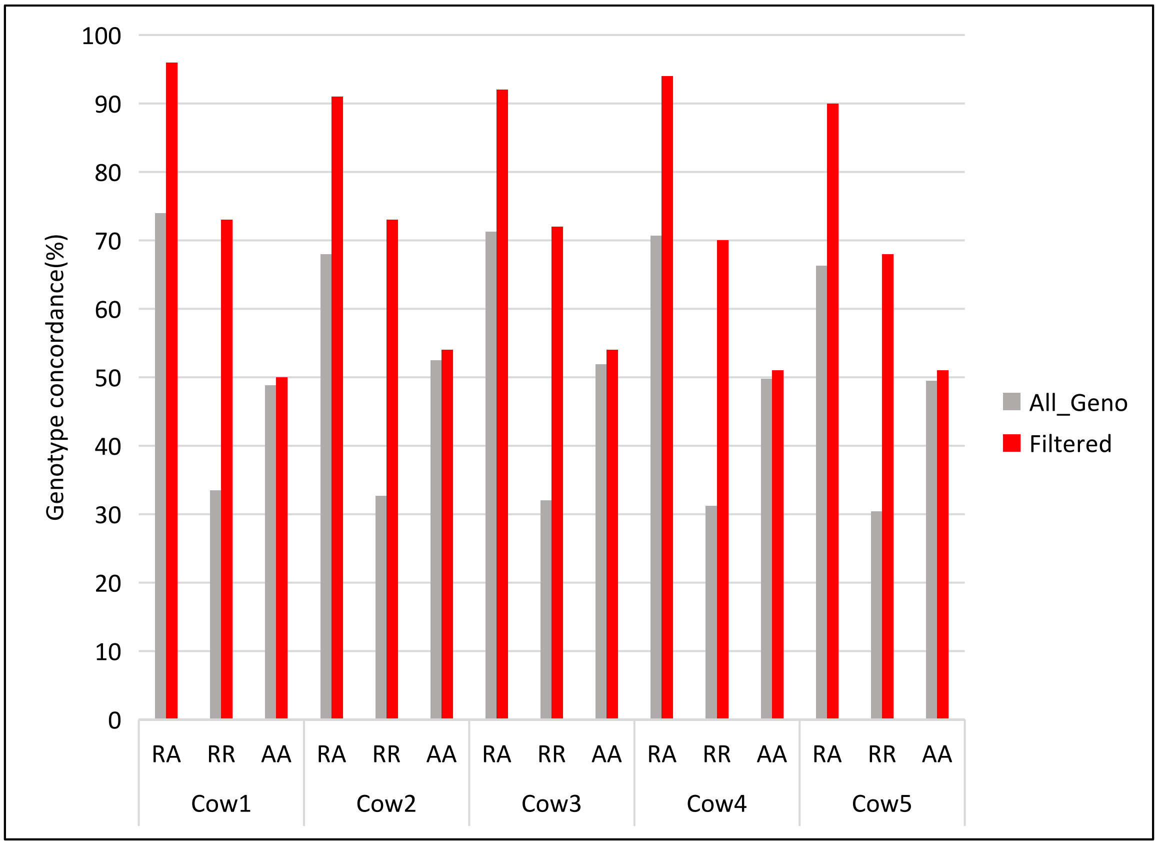

Figure S2 Performance of GBS at genotype level for scenario 1 and scenario 2. Scenario 1 (All geno) indicates all genotypes were considered for evaluation and scenario 2 (filtered) refers to only high-quality filtered genotypes were considered for evaluation. Further details are obtained in Additional file 1: Notes 1. The three states of genotypes were coded as heterozygotes (RA), homozygous (RR) which are reference based and homozygous variants (AA).

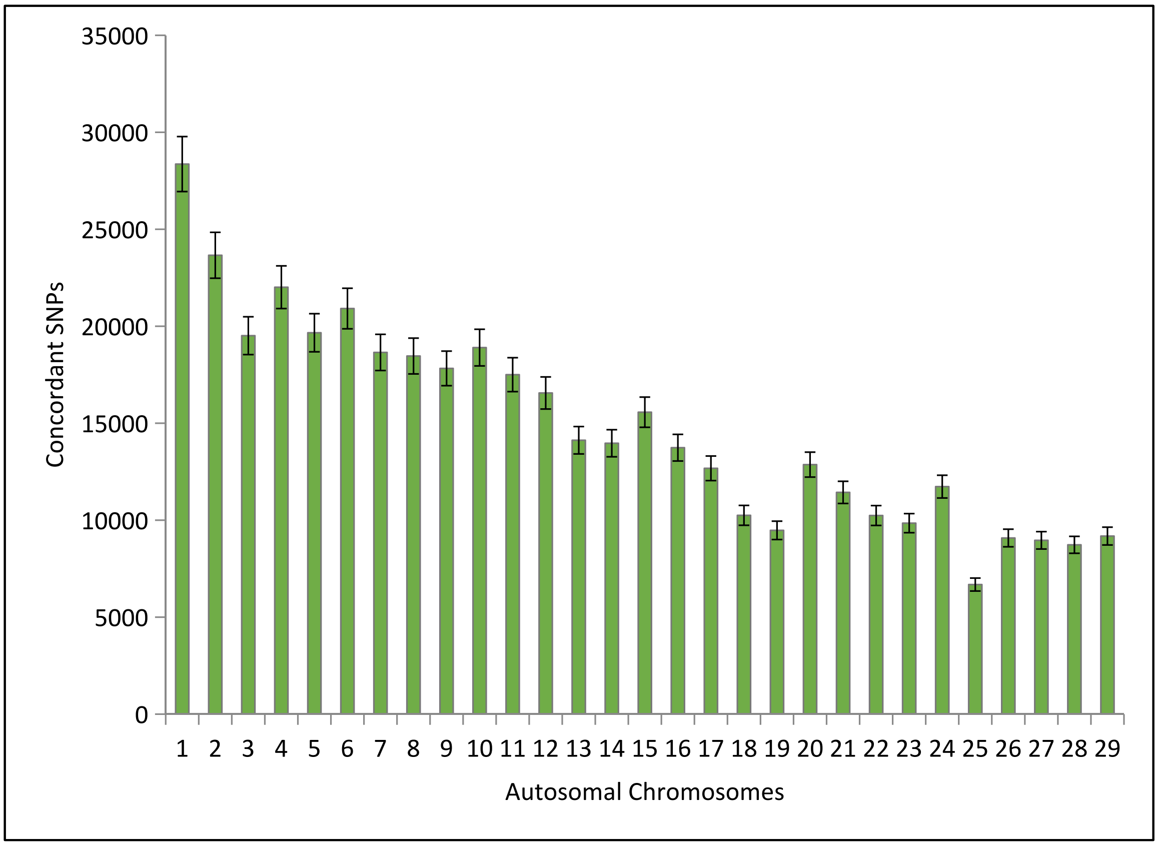

Figure S3 The number of concordant SNPs between GBS and WGS in each chromosome with an average of 99.99% concordance.

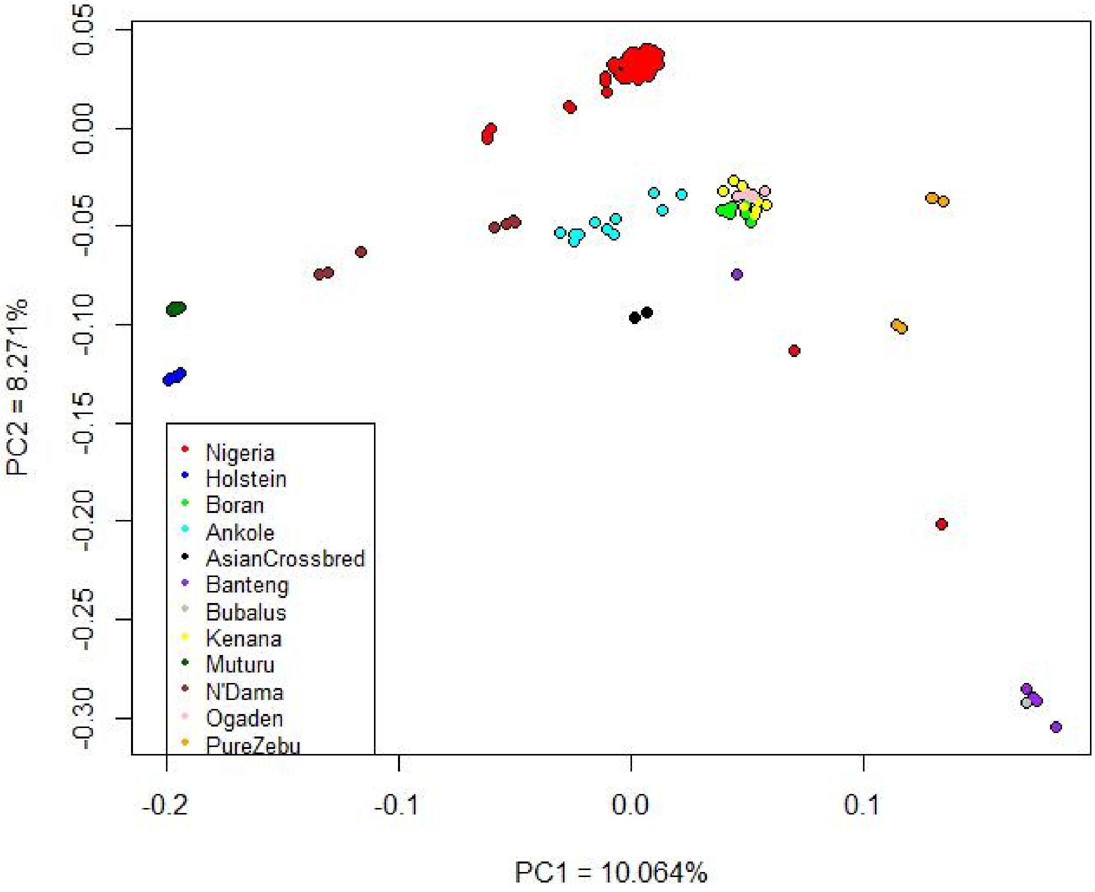

Figure S4 PCA plot of bovines SNP data plotted by using R software. PCs plot for all of the 268 bovine samples based on our data and previous data used in this study [20-23]. Colours represent cattle populations from different geographical regions as described in Supplementary Tables S1 and 2. Nigerian cattle - red, Boran - green, Ankole - cyan, Asian Cross bred - black, Banteng (*B. javanicus*)- purple, *B. bubalis* - grey, Kenana - yellow, Muturu - dark green, N'Dama - brown, Ogaden - pink, Pure zebu from Asia - orange.

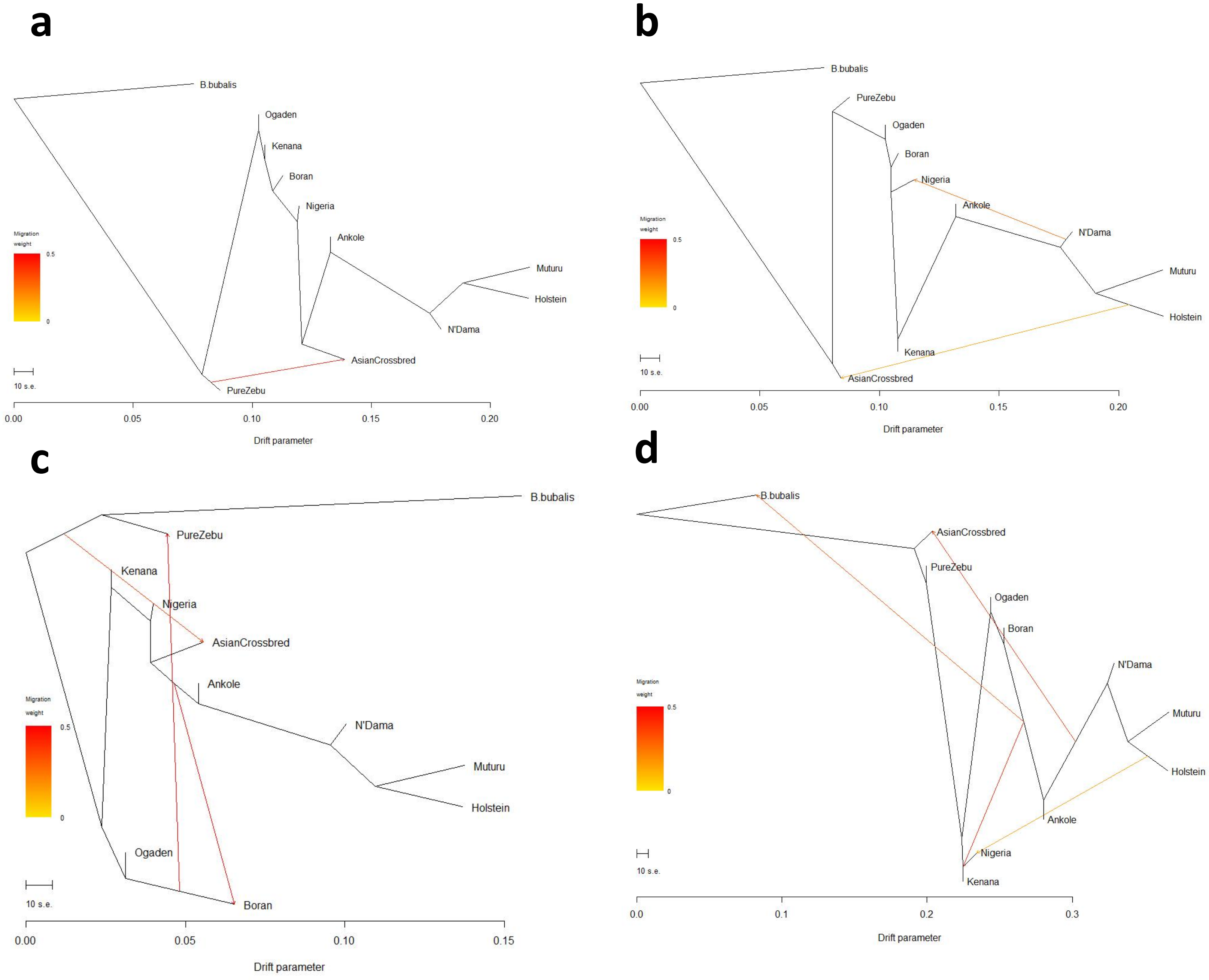

Figure S5 TreeMix analyses depicting evidence of gene flow. Migration patterns were run at four different migration edges using -m parameter for m = 1 (a) m = 2 (b) m = 3 (c) and m =4 (d). *B. bubalis* is the outgroup.

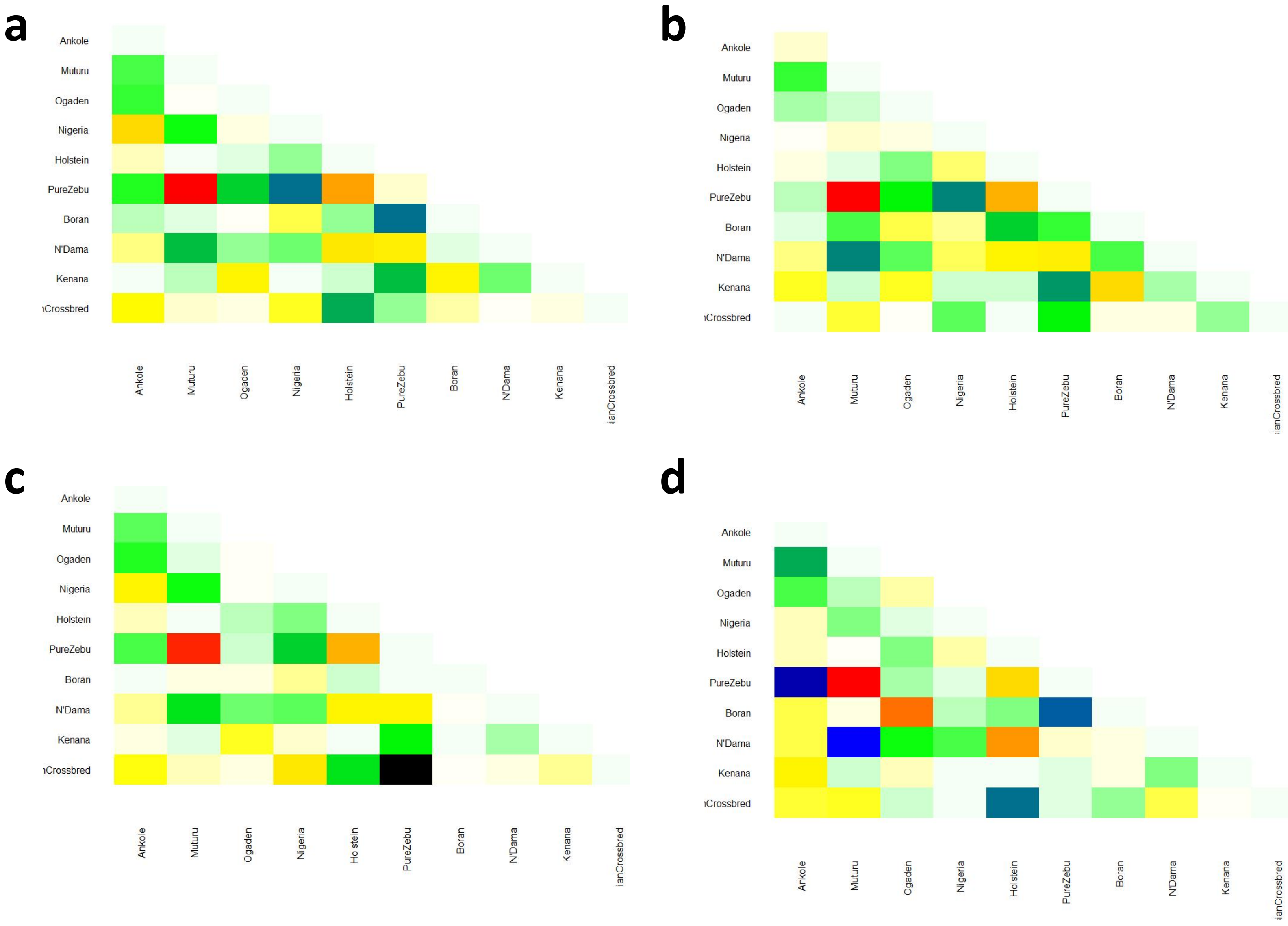

Figure S6 Continuation of the TreeMix analyses showing the residual matrix of the population pairs in the four runs of the migration edges (m1 to 4 in a to d, respectively). We noticed that, TreeMix residuals did not indicate the SE that infers the best fit of the respective pair of populations. This is probably because of the differences in genotyping platforms between WGS and its counterpart GBS data which is associated with missing information due to its low sequencing coverage. Therefore, we recommend that in population genomics studies, datasets generated from different platforms should not be used especially in genome-wide investigations, or else the datasets of both the target population and the reference samples should be consistently genotyped from the beginning of the study prior to downstream analyses.

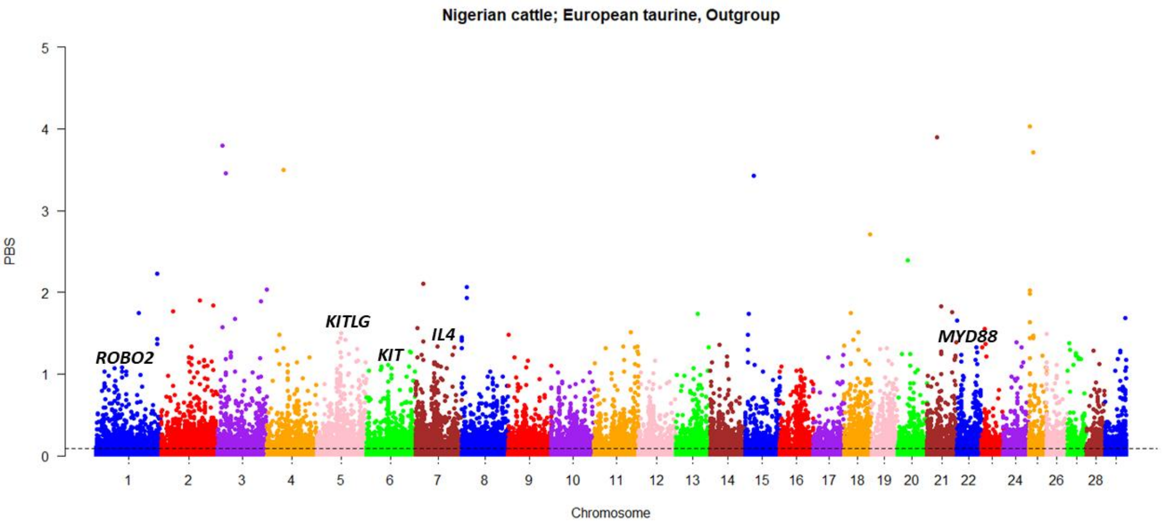

Figure S7 Manhattan plot for PSGs detected by PBS following Nigerian cattle separation from the common ancestor with European cattle. The horizontal black dotted line represents the 1% threshold level.

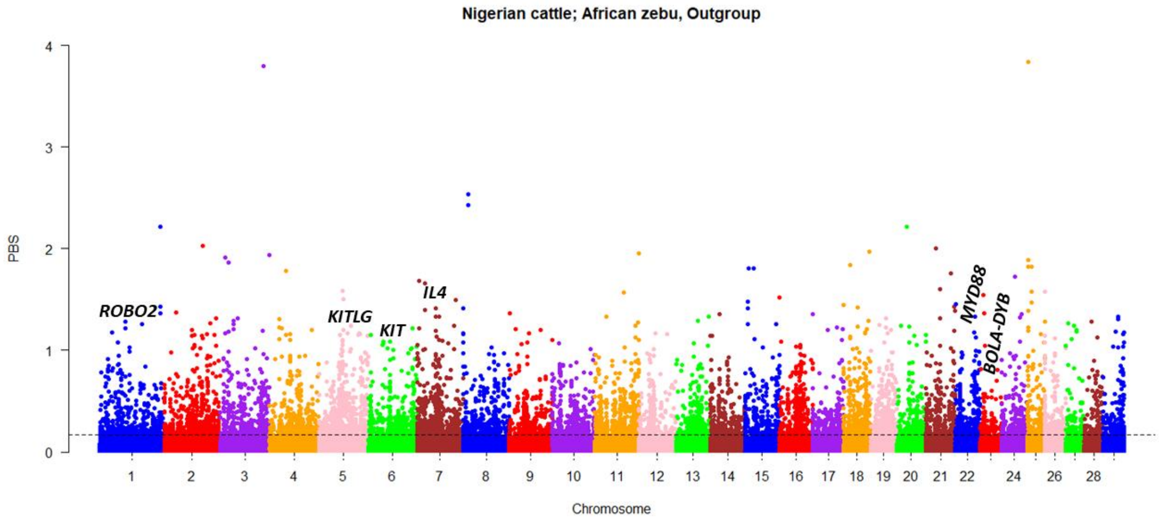

Figure S8 Manhattan plot for PSGs detected by PBS when comparing Nigerian cattle against other African zebu cattle. The horizontal black dotted line represents the 1% threshold level.

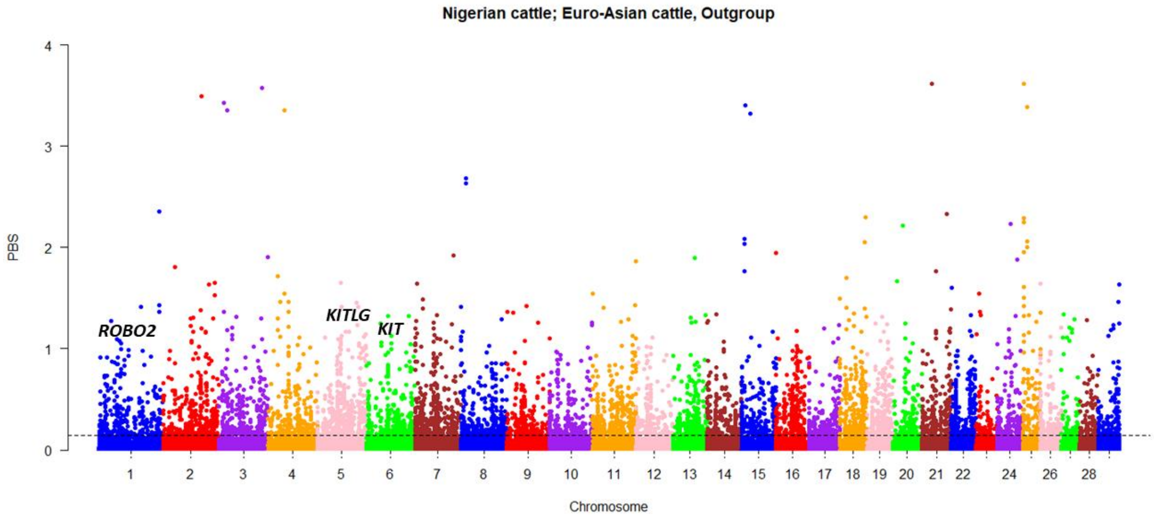

Figure S9 Manhattan plot for PSGs detected by PBS by comparing Nigerian cattle against cattle from both Europe and Asia (Euro-Asian). The horizontal black dotted line represents the 1% threshold level.

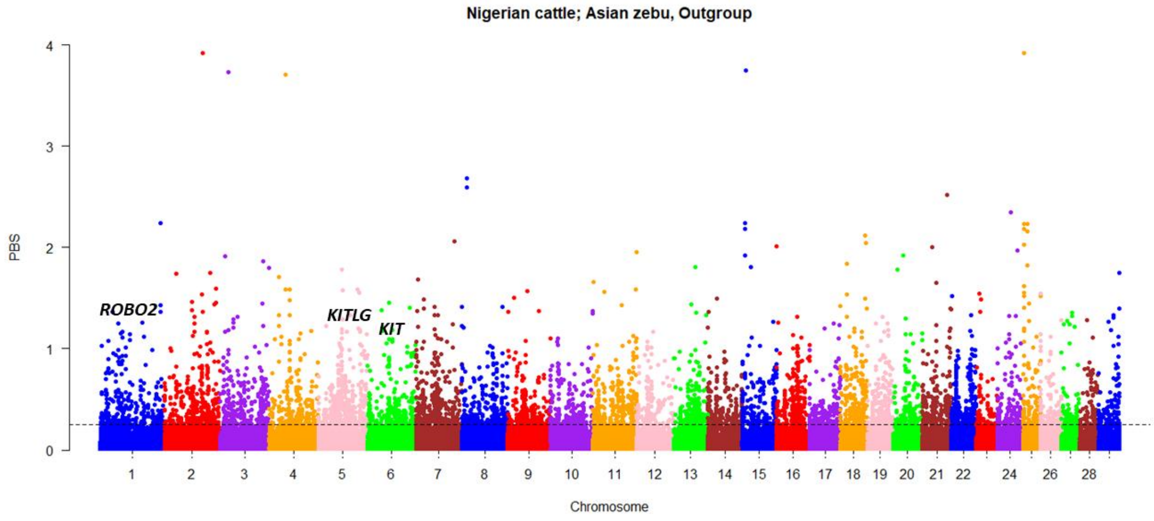

Figure S10 Manhattan plot for PSGs detected by PBS following Nigerian cattle separation from the common ancestor with Asian cattle. The horizontal black dotted line represents the 1% threshold level.

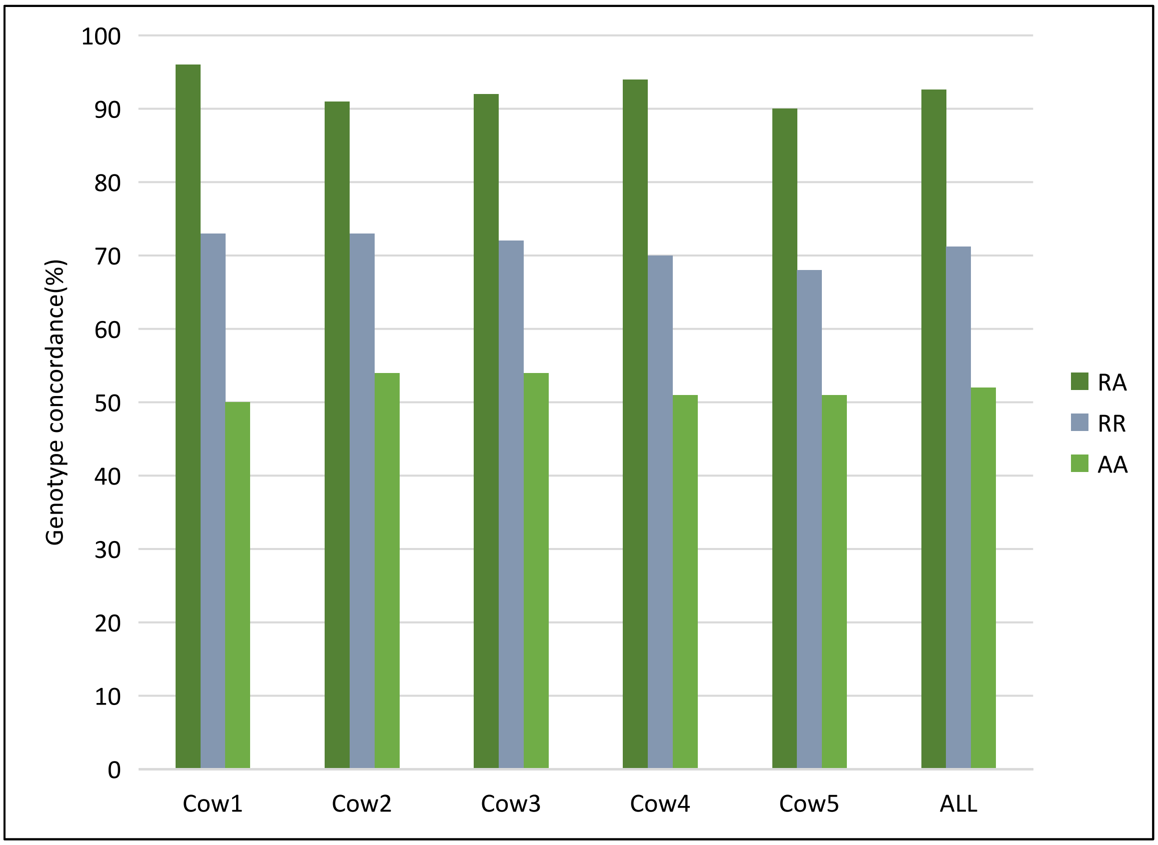

Figure S11 The overall concordance rate (ALL) and that of each genotype in each separate sample for all filtered genotypes (scenario 2). The three states of genotypes were coded as heterozygotes (RA), homozygous (RR) which are reference based and homozygous variants (AA).

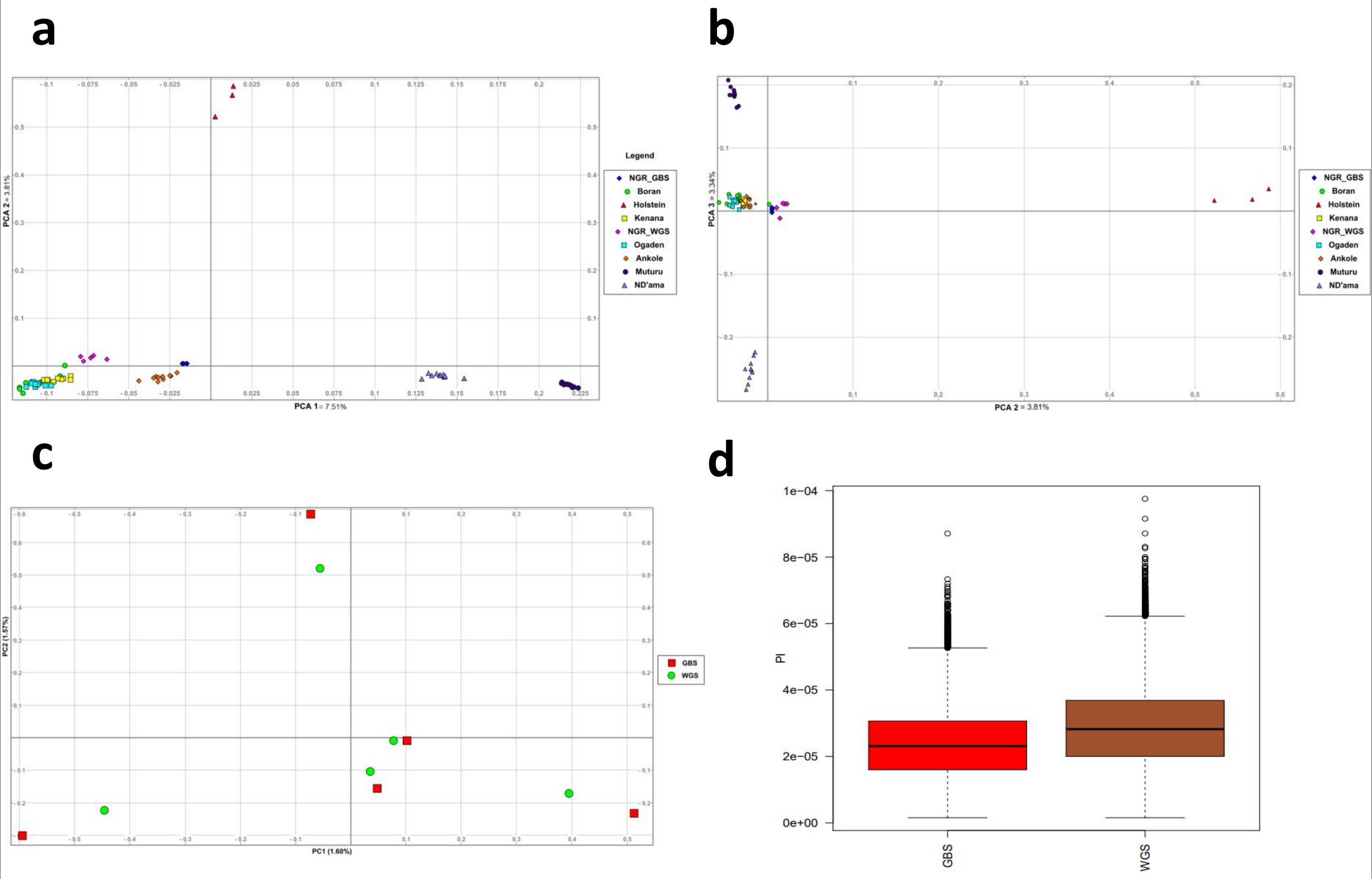

Figure S12 Genetic matrix similarities by using PCA and genetic diversity. Five (5) individuals by both GBS (NGR_GBS) in blue and WGS (NGR_WGS) in pink cluster similarly at PC1 and PC2 (a). Five (5) individuals by both GBS (NGR_GBS) in blue and WGS (NGR_WGS) in pink cluster at the same place at PC2 and PC3 (b). Spatial distribution of the five individuals independent of other populations by GBS (red) and WGS (green) (c). The genetic diversity estimated of the 5 Nigerian cattle individuals sequenced by GBS method (red) and also when re-sequenced by WGS method (brown) (d). The data was initially pruned by Plink v1.9 software.

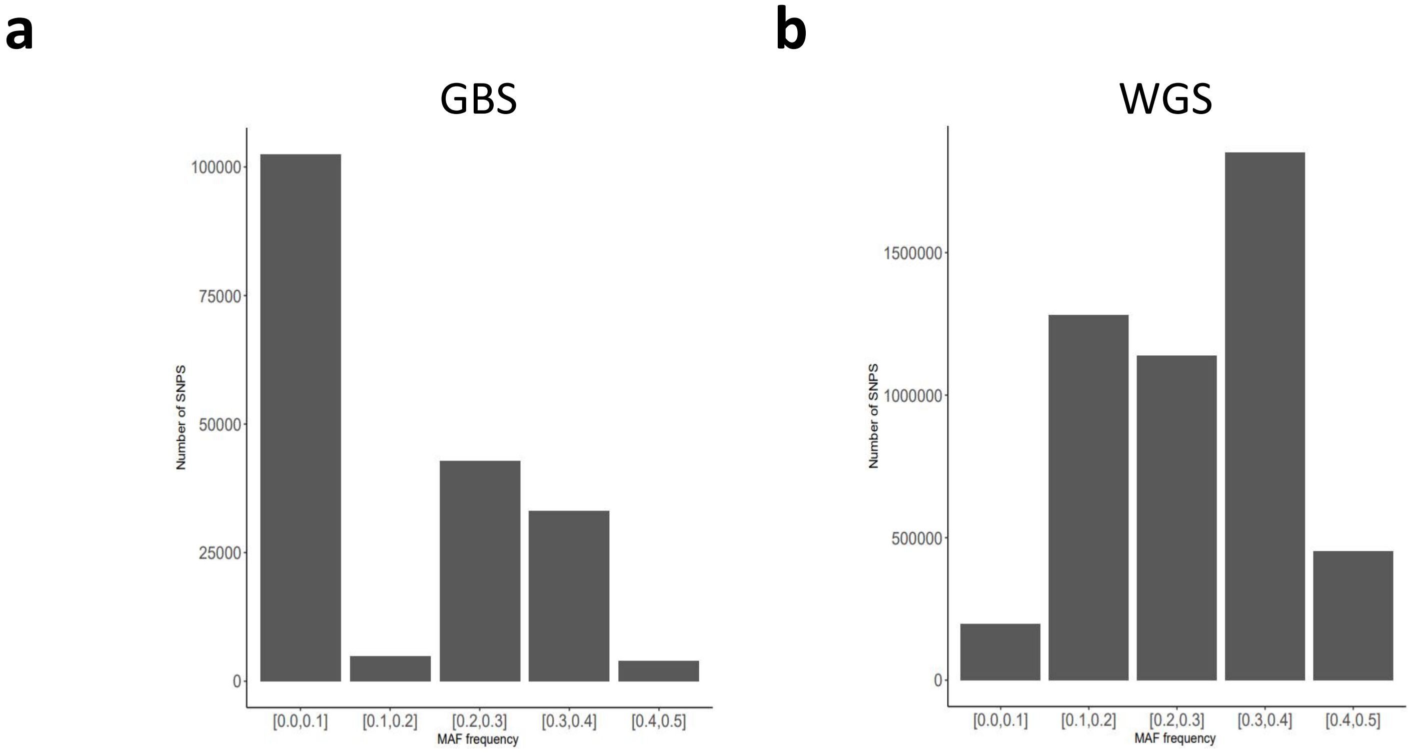

Figure S13 Allele frequency distribution of the 5 Nigerian cattle individuals sequenced by GBS method (a) and also when re-sequenced by WGS method (b). The data was initially pruned by Plink v1.9 software.

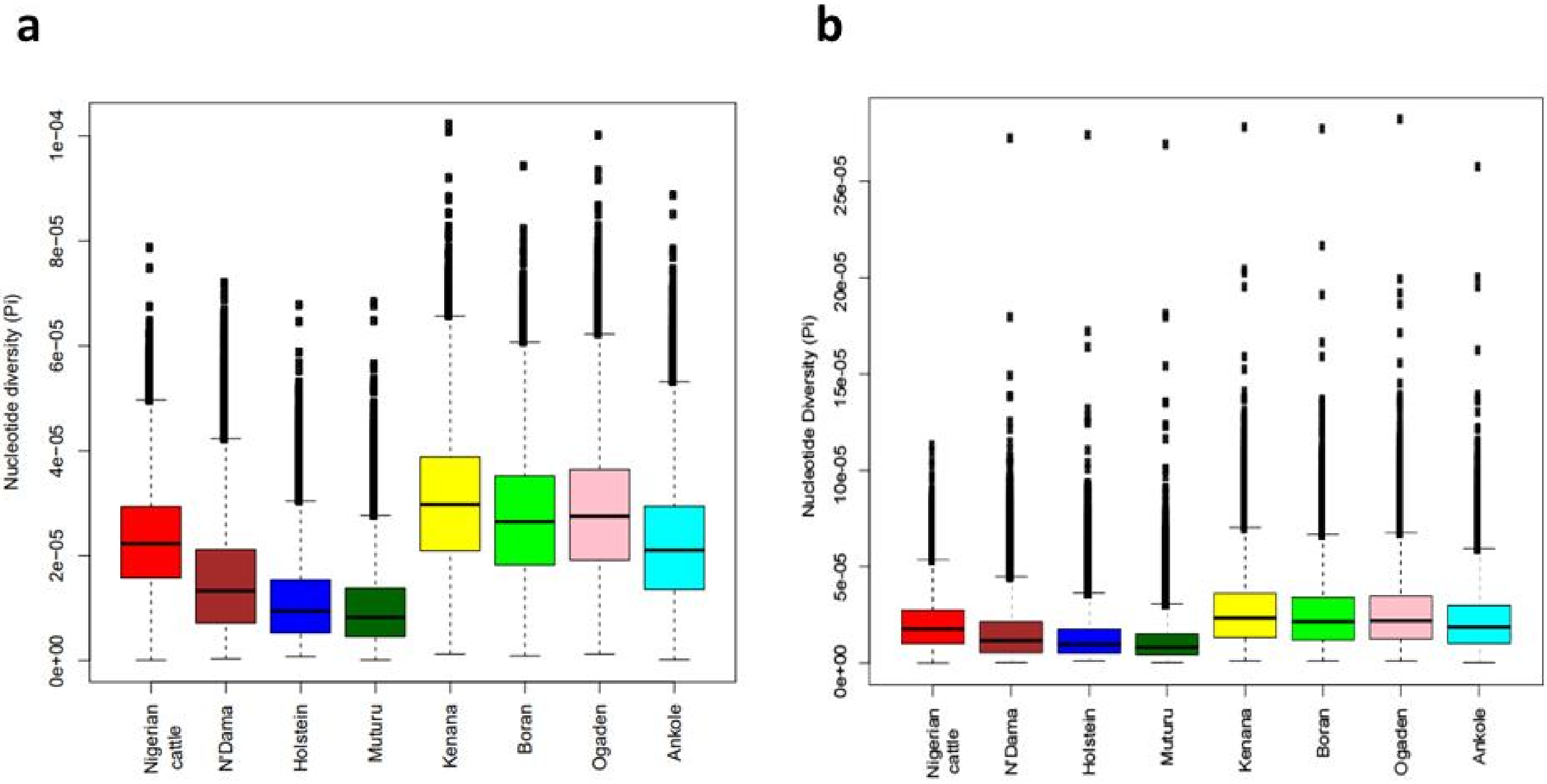

Figure S14 Comparison between versions of the bovine reference genomes. Comparison between versions of the bovine reference genomes. The genetic diversity detected using UMD3.1 reference genome assembly (a) and with ARS-UCD1.2 reference genome assembly (b). The Figure shows reduced nucleotide diversity after lifting over the reference genome. This could imply that the process of lifting over between versions of reference genomes may lose some bit of the genomic content. As in our case we observed that 3% of the genomic content was lost leaving 97% of the total initial genomic content. Our study therefore recommends that calling of the variants should be performed using the same version of reference genome from the very beginning of the analyses.

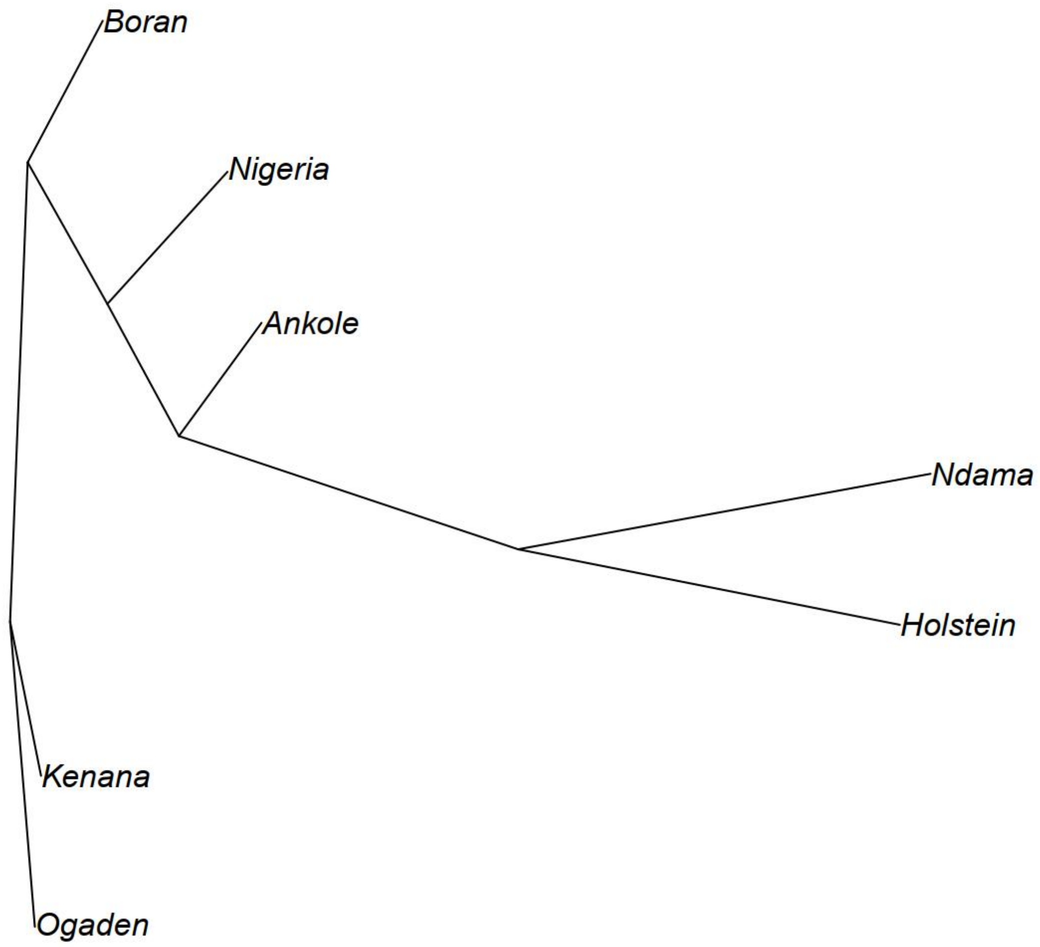

Figure S15 Concatenated phylogenetic tree. The plot was constructed using the five WGS samples randomly selected from the set of 193 Nigerian cattle individuals. Nigerian cattle cluster closely with Ankole and Boran but further apart from the taurine populations.

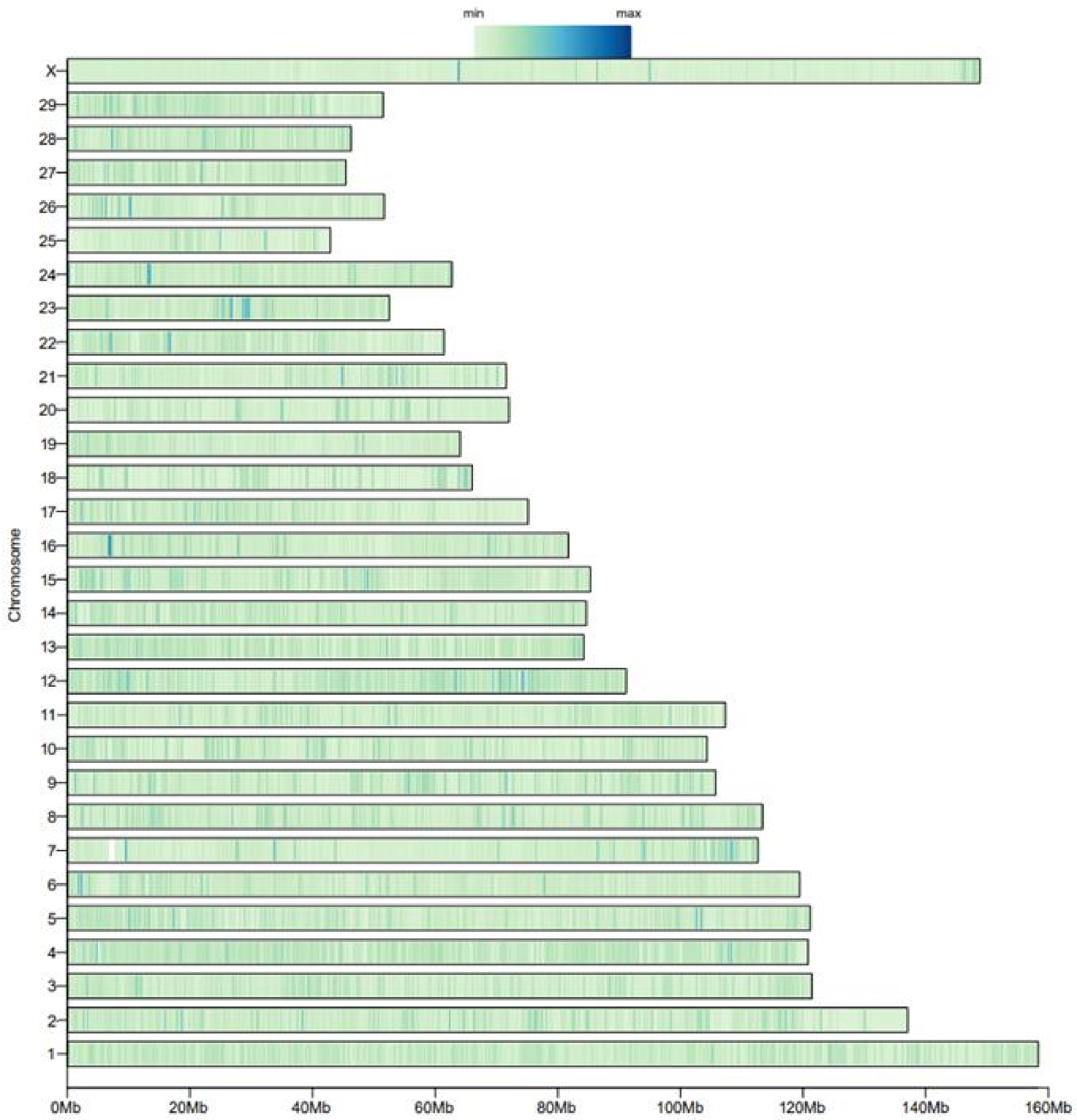

Figure S16 The SNP density in each chromosome within the individual genomes of 193 Nigerian cattle sequenced by GBS approach.

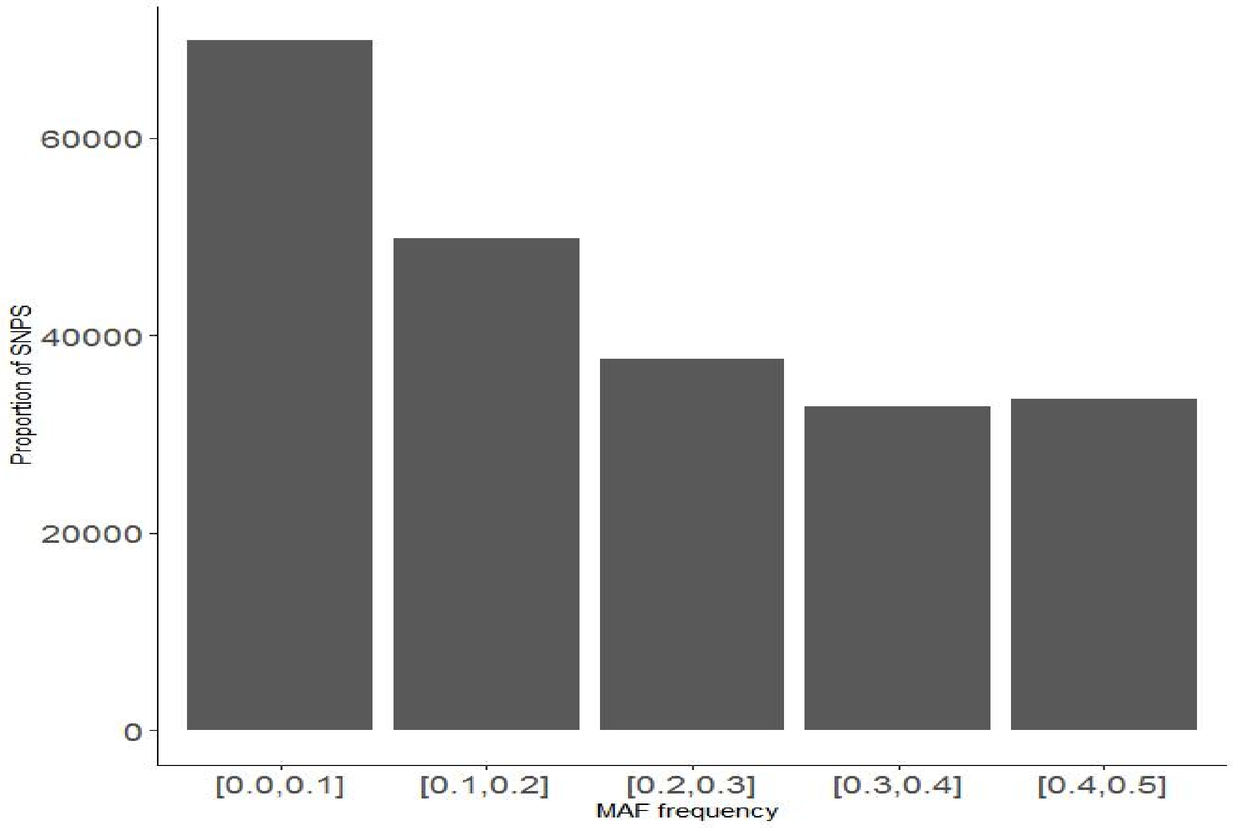

Figure S17 Allele frequency distribution of 193 Nigerian cattle GBS data after stringent pruning by Plink v1.9 software.

Additional file 1: Notes 1 - 2

Notes 1. Comparison between SNP data sets generated by genotyping-by-sequencing and whole-genome sequencing

Genotyping-by-sequencing (GBS) is becoming an interesting commonplace for most researchers in cattle genomics studies; however, its accuracy relative to whole-genome sequencing (WGS) in mammals has not been ascertained. Here we genotyped the genomes of five (5) cattle samples from Nigeria by both platforms and subsequently compared their SNP genotype calls to assess the efficiency of GBS. Usually, variant and/or genotype concordance studies are conducted when two or more different experiments such as genotyping chip arrays and sequencing-based variant callers generate variants with similar alternative alleles at the same loci from the same individual sample(s) for evaluation purposes. We used WGS dataset as our truth set – a gold standard evaluating tool to determine the accuracy and quality of GBS dataset for meaningful downstream analyses in cattle genomic studies. We employed several criteria such as concordance rates at both variant and genotype levels. We further determined the sensitivity and specificity of GBS to ascertain the accuracy of the variants generated against those in our truth set data (WGS dataset). This assessment is important to give insights on the accuracy and quality of GBS SNP calls for accurate and robust conclusions.

Cattle sample information

Our analysis for the evaluation of GBS data was carried out using five samples that were randomly selected from a set of 193 cattle samples from Nigeria. These samples were uniquely

sequenced and genotyped by both GBS pipeline procedures and WGS for comparison (Additional file 1: Figure S1).

SNP discovery and genotyping

These five Nigerian samples were genotyped separately by two different SNP discovering platforms: the GBS SNP discovering platform [1] and the WGS SNP discovering platform. The pair-end reads generated by each respective sequencing library were first mapped separately against the bovine reference genome (UMD 3.1) using BWA *mem* [2] prior to scoring of SNPs and genotyping. Marking and removal of duplicates were done using the Picard tool. Both datasets (GBS and WGS) were subjected to GATK v3.8 SNP detection pipeline and used UnifiedGenotyper tool to call SNPs. The detected raw variants were subjected to hard filtering criteria according to GATK Best Practices recommendations [3-5]. The filtering criteria include the following: "QD < 2.0 || FS > 60.0 || MQ < 40.0 || MQRankSum < -12.5 || GQ < 20 || QUAL < 50.0 || ReadPosRankSum < -8.0 || ((MQ0 / (1.0 * DP)) > 0.1)">" for GBS and the same parameters were applied in WGS data with additional of AB > 0.75 || SB > 0.1 parameters. Further, only 20% of missing information was tolerated in both datasets using VCFtools [6] generating high quality biallelic SNPs of > 95% call rate. To analyze the genotype data, the three states of genotypes were coded as heterozygotes (RA), homozygous (RR) which are reference based and homozygous variants (AA).

The following statistics were used to compare the performance and concordance rates between the GBS and WGS SNP scores: 1) Bi-allelic variants concordance rate and 2) genotype concordance rate 3) Sensitivity and Specificity and 4) The transition to transversion (ti/tv) ratio (Additional file 1: Figure S1) and 5) the correlation test between genetic distances computed by

GBS and WGS for SNPs typed by both methods. Further, we used several terminologies to evaluate the extent of genotype agreement between GBS and WGS. For instance, we considered genotypes that match in common to be termed as “genotype matches”, but those called differently between the two call sets were termed “genotype mismatches”. The rate of allele concordance was extrapolated only by alleles that were called the same in both datasets such that alleles that are called homozygous in one dataset but heterozygous in another were considered mismatch alleles sharing only a single allele in common. All of the statistics were computed by using tools and modules in GATK for both variant and genotype concordance evaluations. Nonetheless, the concordance rates by genotypes called per each sample were computed in two different scenarios (Additional file 1: Figure S2 and Additional file 1: Table S15): Scenario 1. All genotypes (All Geno) – in this scenario all genotypes such as those regarded as of “no genotype calls” and “unavailable genotype calls” were still included in concordance analysis; Scenario 2. Filtered genotypes (Filtered) – in this second scenario, we excluded and filtered out all genotypes called as “no genotype calls” and “unavailable genotype calls”, as such only genotypes of consistent states in both sets were considered.

Notes 2. Extended results on comparison of GBS and WGS

Apart from determining the rate of concordance based on variants, we also evaluated the efficacy of GBS by comparing its genotypes with respect to those found in WGS. Similarly, only the filtered genotypes in scenario 2 (Additional file 1: Figure S2; Additional file 1: Table S15) were used to determine the concordance rates at the genotype level in which the highest rate of concordance was observed in calling heterozygous with an equivalent of 92.8% concordance

(Additional file 1: Table S16 and Additional file 1: Figure S11). On the other hand, although discordances were observed they were still relatively low as compared to the rate of concordances observed (Additional file 1: Table S16). The high rate of discordance/genotype mismatch is in accordance with the fact that most low sequencing coverage SNP panels have a tendency to underscore heterozygous as homozygous because the latter are considered to be easily genotyped [7, 8].

To further validate the accuracy and performance of GBS, we estimated and compared some statistical metrics such as sensitivity and specificity of GBS and WGS SNP calls. We found that the sensitivity of GBS to WGS calls was 1.8% with 100% specificity (Additional file 1: Figure S1). The former rate extrapolates that GBS platform was able to uncover about 2% of the true gold standard variants which were 99.99% concordant. On the other hand, GBS has the power of completely ignoring false positives that may have resulted from sequencing artifacts or reads alignment errors due to its 100% specificity observed. Nonetheless, the ti/tv ratio for GBS was shown to be 2.03 for all known variants but was very low at novel sites (0.8) (Additional file 1: Figure S1). However, the ti/tv for concordant variants and for the WGS calls was reasonably high at ratios of 2.2 and 2.25 respectively. These two ti/tv ratios are similar indicating the validity and high accuracy of GBS calls which further conformed its applicability in downstream genomic analyses.

We lastly measured the accuracy of GBS to WGS using some population genetics parameters such as genetic diversity and principal component analysis (PCA) (Additional file 1: Figure S12). We first explored the distribution of the polymorphic SNPs (usually that minor allele frequency of a particular allele in the population above 1%) for both datasets by observing their minor allele frequency (MAF) distribution. The GBS and WGS data of the same five individuals were

pruned by using PLINK v1.9 software and obtained 187,171 and 4,921,499 total number of variants respectively falling within the category of minor alleles ($0.0 \leqslant$ MAF < 0.5). The variants within this category for GBS data behave by skewing more to the right (Additional file 1: Figure S13a) compared to WGS data (Additional file 1: Figure S13b). The density of SNP variants between 0.0 – 0.1 is seemingly higher in GBS data compared to WGS. Nonetheless, the amount of polymorphic information captured by GBS is observed to decrease disproportionally with the increase in MAF particularly between 0.1 – 0.5 compared to WGS, which could probably indicate that GBS generally underestimates the frequency with regards to WGS. PCA was applied to analyze the spatial distribution of the five individuals in both datasets (Additional file 1: Figure S12a-c). We observed similar pattern of clustering of the five individuals in both datasets, which further raises the level of confidence that GBS data can be applied in downstream genetic analyses. The genetic diversity was also inferred based on their overlapping genomic regions as shown in Additional file 1: Figure S12d. It is shown that the genetic diversity captured by GBS was slightly low compared to WGS probably due to the overall low MAF depicted by GBS. Generally, these parameters have highlighted the differences observed in the allele frequencies estimated by the two different genotyping platforms, and probably the difference is due to artefact brought about by the low sequencing coverage of GBS (~5X). The coverage of GBS is far too low compared to that of WGS which is averagely ~10X (Additional file 2: Table S14)

Overall, we show that GBS approach is generally applicable in downstream cattle genomic analyses due to its high concordance with the WGS data that were generated using the same cattle samples based on the variant calling information. The genotypes assessment showed that GBS had a ~93% rate of concordance for heterozygous calls, referring to having the same

heterozygosity calls at the same site in both datasets along the genotype field, despite of some inadequacy in genotype counts (Additional file 1: Table S16) The low proportion (2.6%) of the called heterozygous genotypes by GBS detected in WGS is obviously due to the low sequencing coverage of GBS (Additional file 1: Table S16). Some studies indicate that to generate heterozygous calls in diploid organisms such as cattle requires a minimum amount of sequence coverage reads probably above 5X, below of which may lead to inadequate heterozygous calls [9]. Such cases are mostly observed in low sequencing coverage panels (below 5X) such as that observed in this study. However, studies have shown that this situation could be overcome by selecting the representative individuals of the putative genetic variation or through genotyping a large number of individuals.

Nevertheless, the low sequencing coverage of GBS is not regarded as a limitation factor in acquisition of accurate genotypic information for meaningful biological assessment even if imputation is not applied [8]. According to the transition to transversion ratio, sensitivity and specificity statistical metrics have shown high accuracy of GBS calls for meaningful biological downstream analyses. However, the low value of ti/tv observed for specific novel sites indicate that these sites maybe associated with false positives calls as a result of sequencing errors reflecting low degree of accuracy in these sites.

Because of high concordance rates observed, our study concludes that GBS generates enough polymorphic information that could be used in cattle genomic studies (for example in population genetics) regardless of its low sequencing read depth (a factor for a reduced overall cost of sequencing per sample) compared to WGS which is very expensive due to its high sequencing reads coverage. This discrepancy however, can be minimized by genotyping a large number of individuals so as to call enough variants especially those located at rare loci [10]. Usually, low

sequence coverage datasets contain minimal levels of polymorphic information (Additional file 1: Figure S13a) as compared to when more individuals are included for genotyping (Additional file 1: Figure S17). Not only that, but also, the use of imputation helps improve the quality of genotypes called in low sequence coverage datasets [8, 11]. We therefore recommend that GBS can be applied in surrogate to WGS, only when involving a large number of individuals, which could be followed by imputation procedures, provided stringent filtering parameters are adhered prior to conducting subsequent downstream analyses.

Additional file 1: Tables S2 - 3

Table S2. Types of cattle samples downloaded from public databases

**Breed**	**Sample size**	**Geographical origin**	**Genetic lineage**	**Reference**	**Database source**
Ankole	12	East Africa	*B. taurus*	Kim *et al*., 2017; Chen *et al*., 2018	PRJNA312138; PRJNA379859
Asian crossbred	3	Asia and East Africa	*B. taurus x indicus*	Kim *et al*., 2017	PRJNA312138
Pure Asian zebu	5	Asia	*B. indicus*	Kim *et al*., 2017	PRJNA312138
Boran	10	East Africa	*B. indicus*	Kim *et al*., 2017	PRJNA312138
Holstein	7	Europe	*B. taurus*	Lee *et al*., 2014	PRJNA210521
Kenana	8	East Africa	*B. indicus*	Kim *et al*., 2017	PRJNA312138
Muturu	8	West Africa	*B. taurus*	Tijjani *et al*., 2019	PRJNA386202
N'Dama	7	West Africa	*B. taurus*	Kim *et al*., 2017	PRJNA312138
Ogaden	9	East Africa	*B. indicus*	Kim *et al*., 2017	PRJNA312138
Banteng	5	Asia	*B. javanicus*	Chen *et al*., 2018	PRJNA379859
Water buffalo	1	Asia	*B. bubalis*	Chen *et al*., 2018	PRJNA379859
**Total**	**75**				

Table S3 Introgression analysis and evaluation of genetic affinity using *D*-statistics test calculated by a phylogenetic tree (Outgroup (P3(P1, P2))). The outgroup in all comparisons is the *Bos javanicus* (Banteng). Statistical significance is evaluated using a two-tailed *Z* test as at -3 ≤ Z ≥ 3 showing evidence of gene flow and introgression. Significant tests are shown in bold and italicized-bolded those involving Nigerian cattle.

P1	P2	P3	Outgroup	*D*	SD	SE	*Z*	*D*-statistics
NC	**EAZ**	**EUT**	Banteng (*B. javanicus*)	**0.0282**	0.458	0.0091	**3.089**	**ABBA**
**EAZ**	NC	**EUT**	Banteng (*B. javanicus*)	-0.0282	0.458	0.0091	**-3.089**	**BABA**
**EUT**	NC	**EAZ**	Banteng (*B. javanicus*)	-0.0646	0.61	0.0121	**-5.313**	**BABA**
**EUT**	EAZ	**NC**	Banteng (*B. javanicus*)	***-0.0364***	0.5889	0.0117	***-3.106***	***BABA***
**AFT**	EAZ	**NC**	Banteng (*B. javanicus*)	***-0.0806***	0.4637	0.0092	***-8.725***	***BABA***
**AFT**	NC	**EAZ**	Banteng (*B. javanicus*)	-0.0975	0.5059	0.0101	**-9.675**	**BABA**
EAZ	NC	AFT	Banteng (*B. javanicus*)	-0.0171	0.4482	0.0089	-1.91	BABA
NC	EAZ	AFT	Banteng (*B. javanicus*)	0.0171	0.4482	0.0089	1.91	ABBA
ASZ	**EAZ**	**NC**	Banteng (*B. javanicus*)	***0.2095***	0.2709	0.0054	***38.808***	***ABBA***
ASZ	**NC**	**EAZ**	Banteng (*B. javanicus*)	***0.1097***	0.3843	0.0077	***14.325***	***ABBA***
**EAZ**	NC	**ASZ**	Banteng (*B. javanicus*)	-0.1021	0.3649	0.0073	**-14.052**	**BABA**
NC	**EAZ**	**ASZ**	Banteng (*B. javanicus*)	0.1021	0.3649	0.0073	**14.052**	**ABBA**

*Note: NC = Nigerian cattle; EAZ = East African zebu; EUT = European taurine; ASZ = Asian zebu; AFT = African taurine; P1-P3 = Populations; SD = Standard deviation; SE = Standard error

Additional file 1: Tables S15 – 16

Table S15 Concordance rate by genotypes called per each sample expressed in percentage in two different scenarios: 1. All genotypes - include no genotype calls and unavailable genotype calls; 2. Filtered genotypes - exclude no genotype calls and unavailable genotype calls

	**Genotype counts**					
	**All genotypes**			**Filtered genotypes**		
**Sample ID**	**RA**	**RR**	**AA**	**RA**	**RR**	**AA**
cow_1	21199	707415	178545	16335	323622	173686
cow_2	13699	676974	190315	10231	304884	183464
cow_3	23321	683163	189282	18147	304012	182968
cow_4	16435	686642	190567	12388	307230	185713
cow_5	17390	684990	195564	12776	304730	189608
**Total**	92044	3439184	944273	69877	1544478	915439
**Average Concordance rate**	**70.06%**	**31.96%**	**50.50%**	**92.80%**	**71.20%**	**52.10%**

Table S16 The proportion of Genotype matches (bold) and genotype mismatch calls (un-bold) between GBS and WGS from the filtered genotypes in Table 1 (scenario 2).

		**WGS**			
		RR	RA	AA	Proportion called *wt WGS
	RR	**71.2**	28.1	0.7	43.5
**GBS**	RA	3.9	**92.8**	3.3	2.6
	AA	1.2	46.7	**52.1**	18.9

Note: *wt ~ with respect to

References

1. Elshire RJ, Glaubitz JC, Sun Q, Poland JA, Kawamoto K, Buckler ES, Mitchell SE. A robust, simple genotyping-by-sequencing (GBS) approach for high diversity species. PLoS One. 2011;6:e19379.

2. Li H. Aligning sequence reads, clone sequences and assembly contigs with BWA-MEM. 2013.

3. DePristo MA, Banks E, Poplin R, Garimella KV, Maguire JR, Hartl C, Philippakis AA, del Angel G, Rivas MA, Hanna M et al. A framework for variation discovery and genotyping using next-generation DNA sequencing data. Nat Genet. 2011;43:491-498.

4. McKenna A, Hanna M, Banks E, Sivachenko A, Cibulskis K, Kernytsky A, Garimella K, Altshuler D, Gabriel S, Daly M et al. The Genome Analysis Toolkit: a MapReduce framework for analyzing next-generation DNA sequencing data. Genome Res. 2010;20:1297-1303.

5. Van der Auwera GA, Carneiro MO, Hartl C, Poplin R, Del Angel G, Levy-Moonshine A, Jordan T, Shakir K, Roazen D, Thibault J et al. From FastQ data to high confidence variant calls: the Genome Analysis Toolkit best practices pipeline. Curr Protoc Bioinformatics. 2013;43: 11.10.1-11.10.33.

6. Danecek P, Auton A, Abecasis G, Albers CA, Banks E, DePristo MA, Handsaker RE, Lunter G, Marth GT, Sherry ST et al. The variant call format and VCFtools. Bioinformatics. 2011;27:2156-2158.

7. Darrier B, Russell J, Milner SG, Hedley PE, Shaw PD, Macaulay M, Ramsay LD, Halpin C, Mascher M, Fleury DL et al. A Comparison of Mainstream Genotyping Platforms for the Evaluation and Use of Barley Genetic Resources. Front Plant Sci. 2019;10:544.

8. Gorjanc G, Cleveland MA, Houston RD, Hickey JM: Potential of genotyping-by-sequencing for genomic selection in livestock populations. Genet Sel Evol. 2015;47:12.

9. Nielsen R, Paul JS, Albrechtsen A, Song YS. Genotype and SNP calling from next-generation sequencing data. Nat Rev Genet. 2011;12:443-451.

10. Druet T, Schrooten C, de Roos AP. Imputation of genotypes from different single nucleotide polymorphism panels in dairy cattle. J Dairy Sci. 2010;93:5443-5454.

11. Hickey JM. Sequencing millions of animals for genomic selection 2.0. J Anim Breed Genet. 2013;130:331-332.

20. Kim J, Hanotte O, Mwai OA, Dessie T, Bashir S, Diallo B, Agaba M, Kim K, Kwak W, Sung S et al. The genome landscape of indigenous African cattle. Genome Biol. 2017;18:34.

21. Lee HJ, Kim J, Lee T, Son JK, Yoon HB, Baek KS, Jeong JY, Cho YM, Lee KT, Yang BC et al. Deciphering the genetic blueprint behind Holstein milk proteins and production. Genome Biol Evol. 2014;6:1366-1374.

22. Chen N, Cai Y, Chen Q, Li R, Wang K, Huang Y, Hu S, Huang S, Zhang H, Zheng Z et al. Whole-genome resequencing reveals world-wide ancestry and adaptive introgression events of domesticated cattle in East Asia. Nat Commun. 2018;9:2337.

23. Tijjani A, Utsunomiya YT, Ezekwe AG, Nashiru O, et al. Genome sequence analysis reveals selection signatures in endangered trypanotolerant West African Muturu cattle. Front Genet. Front Genet. 2019;10:442.
